# PROTAC-mediated degradation of Bcl-xL potentiates target therapy in preclinical melanoma models

**DOI:** 10.1186/s13046-025-03635-w

**Published:** 2026-01-08

**Authors:** Elisabetta Valentini, Giulia Gentile, Marta Di Martile, Simona D’Aguanno, Matteo Brignone, Adriana Maria Di Stefano, Marica Di Caprio, Elisa Melucci, Claudio Botti, Fabio Pelle, Arianna Ortolano, Luigi Fattore, Rita Mancini, Gennaro Ciliberto, Dante Rotili, Donatella Del Bufalo

**Affiliations:** 1 Preclinical Models and New Therapeutic Agents Unit, IRCCS Regina Elena National Cancer Institute, Via Elio Chianesi 53, Rome, Italy; 2 Department of Medical Biotechnologies, Policlinico Le Scotte, University of Siena, Siena, Italy; 3 Pathological Anatomy Unit, IRCCS Regina Elena National Cancer Institute, Rome, Italy; 4 Breast Surgery Unit, IRCCS Regina Elena National Cancer Institute, Rome, Italy; 5 Department of Anatomy, Histology, Forensic- Medicine and Orthopedics, Sapienza University of Rome, Rome, Italy; 6 Department of Life Science, Health, and Health Profession, Link Campus University, Rome, Italy; 7 Scuola Superiore di Studi Avanzati Sapienza, Sapienza University of Rome, Rome, Italy; 8 Department of Clinical and Molecular Medicine, Sant’ Andrea Hospital, Sapienza, Italy; 9 Scientific Direction, IRCCS Regina Elena National Cancer Institute, Rome, Italy; 10 Department of Science, Roma Tre University, Rome, Italy

**Keywords:** Melanoma, Bcl-xL, PROTAC, DT2216, Target therapy

## Abstract

**Background:**

Bcl-xL plays an important role in tumors from different origins, including melanoma, and for this reason it has been widely targeted with small-molecule BH3 mimetics, which unfortunately show several adverse effects. To overcome this limitation, selective Bcl-xL proteolysis-targeting chimera degraders have been developed. Among these, DT2216, a candidate in phase I/II clinical trials, has demonstrated antitumoral activity in preclinical cancer models from different origins, not including melanoma.

**Methods:**

By using several established and patient-derived BRAF *wild type* and mutated melanoma cells, we performed western blot analysis and MTT assay to study DT2216 effect on Bcl-xL protein levels and cell viability, respectively. Combination studies were performed on BRAF mutated melanoma cells treated with DT2216 and Dabrafenib/Trametinib or on *wild type* melanoma cells treated with DT2216 and Trametinib or S63845. Combination index was calculated to study drug interactions. Apoptotic induction was studied through western blot (PARP-1 cleavage), cytofluorimetric (subG1 peak in the cell cycle) and live-cell fluorescent imaging of activated caspases 3/7 analyses. Group differences were analysed with a two-sided paired or unpaired Student’s t-test. To investigate the effect of the combination treatment in vivo, A375luc melanoma cells were inoculated in xenograft mice, then treated with Dabrafenib/Trametinib or DT2216, alone or in combination, for three weeks. Differences between groups, were analysed with Mann-Whitney test.

**Results:**

DT2216 induced the specific and long-lasting degradation of Bcl-xL protein, and reduced cell viability, in a concentration-dependent manner. Of note, a positive correlation between Bcl-xL degradation and sensitivity to DT2216 was observed, being cells with higher degradation the most sensitive to DT2216. In combination studies, DT2216 was able to enhance the activity of target therapy regardless BRAF mutational status. Moreover, the Mcl-1 specific inhibitor, S63845, potentiated the efficacy of DT2216 in melanoma cells in which DT2216 determined an increase of Mcl-1 protein. Interestingly, DT2216 also increased the activity of target therapy in melanoma cells resistant to Dabrafenib and Trametinib. Finally, experiments in a xenograft mouse melanoma model highlighted DT2216 potentiating effect of target therapy, not only inducing a significant reduction of tumor growth, but also showing a longer disease control.

**Conclusion:**

Our findings provide new insights for combination therapy including Bcl-xL degradation for melanoma treatment.

**Supplementary Information:**

The online version contains supplementary material available at 10.1186/s13046-025-03635-w.

## Background

Metastatic cutaneous melanoma (hereafter melanoma) is considered one of the most aggressive forms of skin cancer. Because of its bad prognosis, melanoma represents an important challenge for research [1]. Mutations of the BRAF gene have been identified as driver for the development of this type of cancer, occurring in about 50% of melanoma patients. They constitutively activate BRAF kinase leading to MAPK pathway activation and, in turn, to uncontrolled cell proliferation.

In the last decades, many efforts have been made to offer new therapeutic options to patients with advanced melanoma. These efforts have led to the development of (i) target therapy, involving BRAF/MEK inhibitors (MAPKi) for patients who carry BRAF mutation, and (ii) immunotherapy based on anti-PD-1/anti-CTLA4 antibodies, for all patients regardless BRAF status. These innovative therapeutic strategies have achieved long-term survival rates of approximately 50% [2]. However, the emergence of resistance, the inadequate response to treatments, and the absence of target therapy to treat BRAF *wild type (wt)* patients represent an important challenge for researchers. Further efforts are required to find new therapeutic opportunities to increase the survival of metastatic patients [3].

The anti-apoptotic proteins belonging to the Bcl-2 family are involved in the control of intrinsic apoptotic pathway. Their aberrant expression is often related to cancer progression, survival and therapeutic resistance [4]. The main anti-apoptotic proteins are represented by Bcl-2, Mcl-1, and Bcl-xL which, besides their role in the regulation of apoptosis, are involved in many other cellular processes, including resistance to therapy, angiogenesis and autophagy, giving them a pro-oncogenic role in several types of cancer, including melanoma [5]. Bcl-xL protein has been found to be upregulated in human cancer from different origins where it promotes carcinogenesis, functions as a driver of tumorigenesis and progression and is associated with resistance to therapy and with a poor prognosis [6–11]. We and others reported on the pivotal role of Bcl-xL protein on tumor progression-associated properties in melanoma [4, 12, 13]. By using both mouse and zebrafish models, association of melanoma-specific Bcl-xL with tumor immune microenvironment has been evidenced by our group [14]. Very recently, also Bcl-xL dependence by senescent melanoma cells has been demonstrated [15].

Based on this evidence, small-molecule BH3 mimetics have been developed to target Bcl-xL and other anti-apoptotic proteins. In particular, both Bcl-xL specific inhibitors such as A-1331852 [16], A-1155463 [16, 17] and WEHI-539 [18] and pan inhibitors blocking several anti-apoptotic proteins, have been reported to induce in vitro and in vivo antitumoral activity. Among the pan inhibitors, Navitoclax (ABT-263), directed against Bcl-xL, Bcl-2 and Bcl-W proteins [18, 19], since 2007 has entered cancer clinical trials as single agent or in combination therapy. In some cases, Navitoclax proved to be safe and showed clinical efficacy. However, most patients experienced thrombocytopenia as the principal side-effect, because platelets depend on Bcl-xL for their survival [19, 20]. To overcome this limitation, one of the therapeutic strategies for cancer proposed by researchers is the degradation of Bcl-xL [21], and several selective Bcl-xL proteolysis-targeting chimera (PROTAC) degraders have been developed [22]. PROTACs are small hetero-bifunctional molecules consisting of a moiety binding the protein of interest (POI) and a portion binding an E3 ubiquitin ligase, covalently joined via a linker. The formation of a ternary complex, consisting of POI, PROTAC and E3 ubiquitin ligase, promotes the poly-ubiquitination of the POI, which is lastly degraded by the ubiquitin-proteasome system [23, 24].

Among PROTACs, the specific degradation of Bcl-xL protein by DT2216 via Von Hippel-Lindau (VHL) E3 ligase, demonstrated antitumoral activity in preclinical cancer models from different origins, not including melanoma [25–28], without inducing thrombocytopenia [29]. In fact, as platelets express very low levels of VHL, DT2216 does not form a stable ternary complex, does not degrade Bcl-xL, and consequently, does not compromise platelet viability [30]. Sensitivity of senescent melanoma cells to DT2216 or other Bcl-xL specific inhibitors, has been also very recently reported [15]. Preclinical findings with DT2216 allowed its progression to phase I/II clinical trials for advanced solid tumors (NCT04886622, NCT06620302) [31].

In this work we investigated the sensitivity to DT2216 of a panel of established and patient-derived human melanoma cells and xenograft mouse melanoma models, as well as DT2216 ability to potentiate the effect of MAPKi. We highlighted, for the first time, that DT2216 increases the efficacy of MAPK inhibitors, both in vitro and in vivo, regardless BRAF status, through induction of apoptotic cell death.

## Materials and methods

### Cell cultures

Human BRAF *wt/*NRAS mutated (Sbcl1, Me4405, Me2/17), BRAF mutated/NRAS *wt* (A375, LOX IMVI, Skmel28, RPMI7951, ME4686), MAPKi resistant (ME4686DR and LOX IMVIDR [LOXDR]) melanoma cell lines, human fibroblasts (HFF) and endothelial cells (EA-HY926) were used. All melanoma cells were maintained in RPMI-1640 (Euroclone, Milan, IT), except Skmel28 and RPMI7951 cells that were maintained in EMEM (Euroclone). HFF and EA-HY926 were cultured in DMEM (Euroclone) complete medium supplemented with 10% inactivated fetal bovine serum (FBS) (Hyclone, Thermo Fisher Scientific, Waltham, MA, USA), 1% L-glutamine (Euroclone) and 100µg/ml penicillin/streptomycin (Euroclone).

ME4686DR and LOXDR cells were obtained exposing ME4686 and LOX IMVI parental cells to increasing concentrations of Dabrafenib (BRAFi) and Trametinib (MEKi) starting from 2nM to 1nM concentrations, respectively. The selection lasted about 2 months until concentrations of 2µM for the BRAFi and 1µM for the MEKi were reached. ME4686DR cells show an IC_50_ ≥ 1µM for Dabrafenib and IC_50_ = 33,3nM for Trametinib, while IC_50_ values of the sensitive counterparts are 4,11nM and 1,23nM for Dabrafenib and Trametinib, respectively. LOXDR cells show an IC_50_ ≥ 1µM for Dabrafenib and IC_50_ ≥ 100nM for Trametinib, while IC_50_ values of the sensitive counterparts are 333nM and 33,3nM for Dabrafenib and Trametinib, respectively. ME4686DR and LOXDR cells were maintained in RPMI-1640 (Euroclone) complete medium in a final concentration of 0.3µM Dabrafenib and 0.03µM Trametinib.

All cell lines were routinely tested for mycoplasma contamination and authenticated within the last 8 months.

### Patient-derived melanoma cells

Patient-derived melanoma cells (Mel2622 and Mel2648) were obtained from metastatic biopsies (lymph nodes) of melanoma patients, provided by Simona Di Martino (Biobank IRCCS-Regina Elena National Cancer Institute, BBIRE), after patients’ written informed consent following the Declaration of Helsinki. The ethical committee of Regina Elena National Cancer Institute of Rome approved the derivation of melanoma cells from patients (# 1441/20). Briefly, tumors were stored in 50ml tubes (Sigma-Aldrich, St. Louis, Missouri, USA) containing sterile Tissue Storage Solution (Miltenyi Biotec, Bologna, IT) for up to 72 h, then were cut into small pieces using a scalpel, mechanically dissociated using the Miltenyi gentleMACS Dissociator (Miltenyi Biotec) and digested for 1 h at 37°C in RPMI-1640 containing 10mg/mL collagenase IV, 650U/mL DNAse I and 500U/mL hyaluronidase (Miltenyi Biotec). Cells were then filtered through a 70μm cell strainer to make a single cells solution and cell viability was assessed by trypan blue exclusion. After dissociation, cells were cultured in DMEM (Euroclone) supplemented with 10% inactivated FBS, 2% Penicillin-Streptomycin, and 1% Glutamine and maintained at 37°C in a 5% CO2 incubator. The molecular features of Mel2622 and Mel2648 cells and melanoma patients from whom the cells were derived are summarized in Tables S1 and S2.

### Reagents preparation and treatments

For in vitro experiments, DT2216 (0.01-80µM, 4–72 h), C3 (10µM, 4–48 h), C5 (10µM, 4–48 h), Dabrafenib (0.1–0.5µM, 48 h), Trametinib (0.01–0.05µM, 48 h), ABT-199 (1–5µM, 48 h), S63845 (1–5µM, 48 h), the pan-caspase inhibitor z-VAD-fmk (z-VAD, 50µM, 48 h) (MedChem, Monmouth Junction, USA) and the proteasome inhibitor MG132 (5µM, 16 h, Sigma-Aldrich) were dissolved in DMSO and further diluted in complete medium for treatments. As control, cells were treated with DMSO at concentrations not affecting cell proliferation (0.1%-0.4%), depending on the highest concentration of DT2216, BH3 mimetics (ABT-199 and S63845) or MAPKi used for each experiment.

For in vivo experiments, DT2216 was dissolved in 10% DMSO, 40% PEG400 (Sigma-Aldrich), 5% Tween80 (Acros Organics, New Jersey, USA), and 45% NaCl (vehicle), while Dabrafenib and Trametinib were suspended in an aqueous mixture of 0.5% hydroxypropyl methylcellulose (Sigma-Aldrich) and 10% DMSO.

### Cell viability assay

MTT assay (Sigma-Aldrich) was used to evaluate viability of melanoma cells after different treatments. In particular, cells were plated in 96-well plates and, after 24 h, exposed to different concentrations of DT2216, Dabrafenib, Trametinib, S63845 and ABT-199. Combination treatment experiments were performed exposing cells to DT2216 or Dabrafenib and Trametinib, alone or in combination for 48 h, or DT2216 and S63845 or ABT-199, alone or in combination for 48 h. Results were reported as “viability of treated cells/viability of control cells (Ctrl)” × 100 and as mean ± SD of three independent experiments. p-values were calculated between single and combination treatments. GraphPad Prism 9.0 software (Dotmatics, Bishop’s Stortford, UK) was used to evaluate the concentration of the drug reducing 50% cell viability (IC_50_). CalcuSyn 2.0 software (Biosoft, Ferguson, MO) was used to evaluate the combination index (CI). Acquisition of resistance to BRAFi and MEKi by melanoma cells (ME4686DR and LOXDR) was evaluated by quantification of ATP, according to Cell Titer-Glo Luminescent Cell Viability assay protocol (Promega, Madison, WI, USA).

### Western blot analyses

Basal levels of Bcl-2, Bcl-xL and Mcl-1 proteins and expression/cleavage of PARP-1 protein in melanoma cells after different treatments, were evaluated by western blot analyses, as previously described [14, 32, 33]. In order to study the induction of apoptosis, experiments of combination were performed for 48 h. Antibodies directed to Bcl-2, Bcl-xL, Mcl-1 (Cell Signaling, Massachusetts, USA), PARP-1 (BD Bioscience, San Jose, CA), α-tubulin (Santa Cruz Biotechnology, Texas, USA) and Hsp72/73 (Sigma Aldrich) were used. Anti-mouse immunoglobulin G-horseradish peroxidase-conjugated antibodies (Thermo Fisher Scientific) were used as secondary antibodies. Images were acquired by Image Lab Software (Bio-Rad, Hercules, CA, USA) using a ChemiDoc System instrument (Bio-Rad). DC_50_ values were obtained treating cell with doses of DT2216 ranging from 0.01µM to 2µM for 24 h, performing the densitometric analysis and then calculating the dose inducing 50% of Bcl-xL protein degradation (CalcuSyn 2.0 software, Biosoft). All densitometric analyses were performed with Image J software version 1.53a (Rasband, W.S., ImageJ, U. S. National Institutes of Health, Bethesda, Maryland, USA, https://imagej.nih.gov/ij/). Values were expressed as fold change of the protein of interest relative to the housekeeping one.

### Cytofluorimetric analyses

Cytofluorimetric analyses were performed to analyse cell cycle distribution and the presence of cells in the subG1 peak, indicative of apoptosis, after exposure of melanoma cells to different treatments (48 h). To confirm induction of apoptosis, the pan caspase inhibitor, z-VAD (50µM), was added to the treatments. After treatments, cells were fixed with ice-cold 70% ethanol for 24 h at 4 °C, washed in PBS buffer and stained with 500µl of PBS containing RNase A (100µg/ml, Sigma-Aldrich) and Propidium iodide (50µg/ml, Sigma-Aldrich) for 30 min in the dark. Flow cytometric analyses were performed using BD Accuri™C6.

### Caspase-3/7 apoptosis assay

In addition to western blot (PARP-1 expression and cleavage) and cytofluorimetric (presence of subG1 peak in cell cycle distribution) analyses, apoptosis was also detected by the caspase-3/7 apoptosis assay. Briefly, 2 × 10^3^ A375, 3 × 10^3^ LOX IMVI or 3 × 10^3^ Sbcl1 cells were seeded into 96-well plates (Corning). After 24 h, cells were exposed to DT2216 and MAPKi alone or in combination and to 2.5µM Caspase-3/7 Apoptosis Assay Reagent Green (IncuCyte, Sartorius, Gottingen, Germany) in the absence or presence of 50µM z-VAD. Cells were, then, placed in the IncuCyte S3 Live-Cell Analysis System (Sartorius) and imaged every 3 h for a total of 48 h. Total Green Object Integrated Intensity (GCUxµm2/Image) were quantified using IncuCyte software and visualized using a yellow signal. Three replicate wells were used for each treatment and experiments were independently repeated three times.

### In vivo experiments

5 × 10^6^ A375luc cells infected with luciferase-encoding lentivirus (pRRLSIN.cPPTLuciferase. WPRE from Addgene, Watertown, Massachusetts, USA) as previously described [34] were subcutaneously injected in the right flank of 6-8-week-old female athymic CD1 nude mice. After 7 days, mice were randomized into different groups (6 mice for each group) and treated for three weeks with Dabrafenib (5mg/kg, oral gavage [o.g.]), Trametinib (0.1mg/kg, o.g.), DT2216 (15mg/kg, intraperitoneally [i.p.]), alone or in combination. Animals were observed daily, and tumor growth was monitored with a calliper (twice a week, calculating tumor volume [mm^3^] as length × width^2^ × π/6), and by bioluminescent imaging (once a week) as previously reported [34]. The signal was detected using the IVIS Spectrum CT (Perkin Elmer, Waltham, MA, USA) and analysed using Living Image software version 4.7.4. Mice were anesthetized, i.p. injected with 75mg/kg D-luciferin, and imaged 10 min after injection. Photon emission was measured in specific regions of interest. Data were expressed as photon/second/cm^2^/steradian. The intensity of bioluminescence was color-coded for imaging purposes. All procedures involving animals and their care were compliant with the Italian Minister of Health (D.lgs 26/2014, 816/2015-PR of 11/08/2015) and received the relative authorization (n° 563/2021-PR. PI Dr Del Bufalo). Institutional Review Boards of both Regina Elena National Cancer Institute and Minister of Health approved all the procedures involving animals (species, quality, number, discomfort/distress/pain, sacrifice).

### Statistical analyses

All the in vitro experiments described were the results of at least three independent experiments performed in triplicate, unless otherwise indicated. The data were expressed as average ± standard deviation (SD). Group differences were analysed with a two-sided paired or unpaired Student’s t-test.

In vivo experiments were repeated twice by using 6 animals for each experimental group. The data were expressed as average ± standard error of the mean (SEM). Differences between groups were analysed with Mann-Whitney test. Statistical analyses were performed with GraphPad Prism 9.0 software (Dotmatics). Sample sizes were chosen based on previously published data [34] to ensure a power of 80% and an alpha level of 5%. No data or animals were excluded from the analyses. Differences were considered statistically significant for *p* < 0.05.

## Results

### DT2216 induces Bcl-xL degradation and reduces cell viability of melanoma cells

**Fig. 1:**
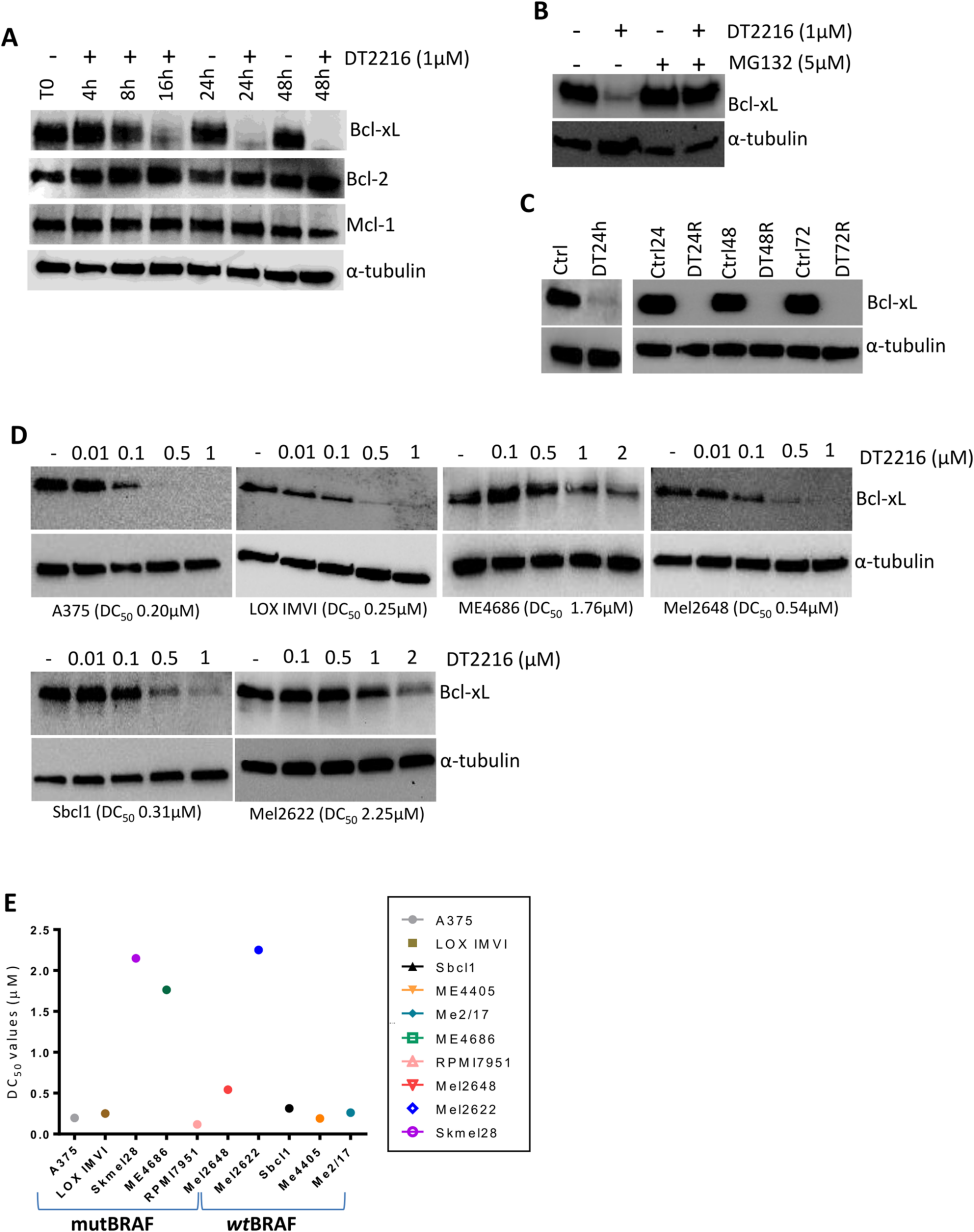
DT2216 degrades Bcl-xL protein in melanoma cells. **A** Western blot analysis of Bcl-xL, Mcl-1 and Bcl-2 proteins expression in A375 cells after treatment with 1µM DT2216 for times ranging from 4 h to 48 h. **B** Western blot analysis of Bcl-xL protein expression in A375 cells after treatment with 1µM DT2216 and 5µM MG132 alone or in combination for 16 h. **C** Western blot analysis of Bcl-xL protein expression in untreated A375 cells at 24-48-72 h of growth (Ctrl24h, Ctrl48h, Ctrl72h) or treated with 1µM DT2216 for 24 h and then maintained in fresh culture medium for 24 h (DT24R), 48 h (DT48R) or 72 h (DT72R). **D** Western blot analysis of Bcl-xL protein expression after treatment with doses of DT2216 ranging from 0.01µM to 1µM (A375, LOX IMVI, Mel2648, Sbcl1 cells) or from 0.1µM to 2µM (ME4686 and Mel2622 cells) for 24 h. BRAF mutated (upper panel) and BRAF *wild type* (lower panel) cell lines were used. Numbers in brackets indicate DC_50_ values. **E** DC_50_ values in all the tested cell lines. A-D Western blot images are representative of two independent experiments with similar results. α-tubulin is shown as loading and transferring control

**Fig. 2:**
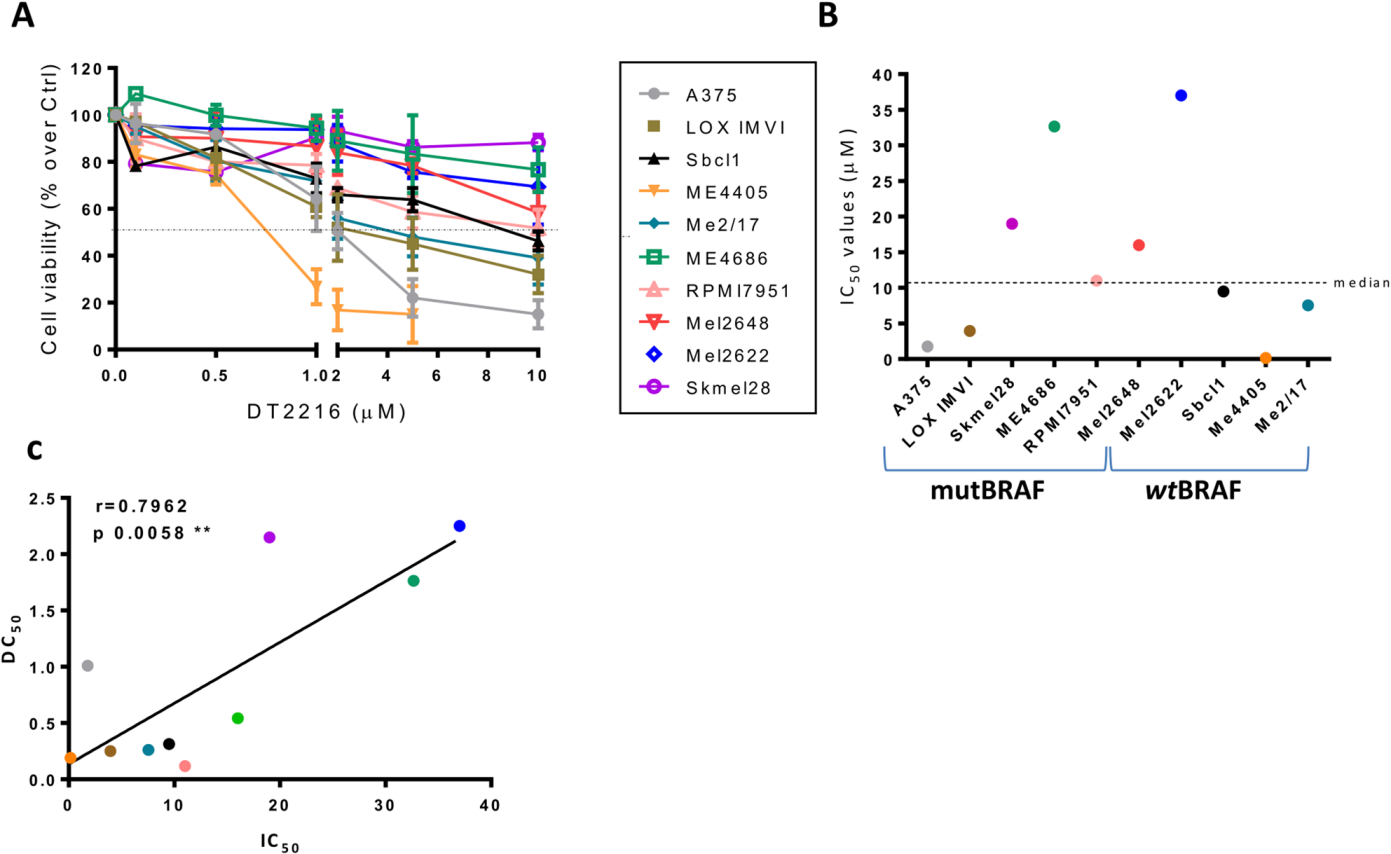
DT2216 reduces cell viability of a panel of melanoma cell lines. **A** Analysis of cell viability of cell lines treated with DT2216 at concentrations ranging from 0.1µM to 10µM for 72 h. Results are reported as “viability of treated cells/viability of control cells (Ctrl)” × 100, and as mean ± SD of three independent experiments. **B** IC_50_ values of melanoma cells treated as reported in (A) calculated from the average of three independent experiments. The dotted line represents the median value of IC_50_. **C** Correlation analysis between IC_50_ (from Fig. 2B) and DC_50_ (from Fig. 1E) values. **p < 0.01. A-C The legend in the middle refers to the cell lines used in all the analyses

Given the relevance of anti-apoptotic proteins belonging to the Bcl-2 family in melanoma progression and response to therapy [4], we first investigated the ability of commercially available PROTACs targeting Bcl-2 (C5), Mcl-1 (C3) [35] or Bcl-xL (DT2216) to degrade their targets in A375 melanoma cells. As shown by western blot analyses, Bcl-xL protein degradation was observed starting from 8 h after exposure to 1µM DT2216, with a complete degradation after 16 h of treatment, while Bcl-2 and Mcl-1 protein expression was not affected, confirming published data [29] reporting DT2216 as specific Bcl-xL degrader (Fig. 1A). Both 10µM C5 (Fig. S1A) and 10µM C3 (Fig. S1B) induced, Bcl-2 and Mcl-1 degradation, starting from 48 h after treatment, respectively. Considering DT2216 higher efficacy in reducing the target protein, we focused our attention on DT2216. A panel of 10 established and patient-derived BRAF *wt* and mutated human melanoma cell lines, all expressing Bcl-xL basal levels (Fig. S1C), were used. Starting from a representative melanoma cell line (A375), we investigated the involvement of the proteasome in DT2216 activity. To this aim, cells were treated with 1µM DT2216 and 5µM proteasome inhibitor MG132 for 16 h. As shown in Fig. 1B, MG132 was able to block the ability of DT2216 to degrade Bcl-xL protein, thus confirming the involvement of the ubiquitin-proteasome system in DT2216 mechanism of action. We next evaluated Bcl-xL protein expression after removal of DT2216 from the culture medium. To this aim, A375 cells were treated with DT2216 for 24 h and subsequently maintained in fresh culture medium in the absence of DT2216 for 24 h, 48 or 72 h. As reported in Fig. 1C, Bcl-xL protein was not detectable after removal of DT2216 at each analysed time-points, thus indicating a persistent degradation of the protein up to 72 h. We next calculated the dose of DT2216 inducing 50% degradation of Bcl-xL protein (DC_50_) in the panel of melanoma cell lines. As shown in Fig. 1D, E and Fig. S1D, DT2216 induced Bcl-xL degradation in all tested cells, even if at different extent, with DC_50_ values ranging from 0.12µM to 2.25µM. As DT2216 activity depends on VHL protein expression, we investigated if the ability of DT2216 to degrade the target protein was linked to the levels of the E3 ligase. As shown in Fig. S1C, all cell lines tested have detectable levels of VHL protein, and no correlation between DC_50_ values and the levels of VHL was observed (r value: 0.1313; *p* value: 0.7176) (Fig. S1E). We next tested the effect of Bcl-xL degradation on melanoma cell viability. To this aim, we treated melanoma cells with DT2216 at concentrations ranging from 0.1µM to 10µM for 72 h. As shown in Fig. 2A, cell viability was differentially affected by DT2216 treatment. As Skmel28, ME4686, Mel2648 and Mel2622 cells were the most resistant to the treatment, they were exposed to higher concentrations of DT2216 ranging from 20µM to 80µM (Fig. S2A). IC_50_ values for all tested cell lines, ranged from 0.16µM to 37µM, being Me4405 the most sensitive and Mel2622 the most resistant cells to the compound (Fig. 2B). Of note, the viability of human immortalized fibroblasts and endothelial cells was not affected by exposure to DT2216 (Fig. S2B). We next investigated the possible correlation between the response of melanoma cells in terms of cell viability (IC_50_) and Bcl-xL degradation (DC_50_). As reported in Fig. 2C, a positive correlation between the two parameters was evidenced (r value: 0.7962; p value: 0.0058). In order to understand if the response of melanoma cells to combinatorial treatments correlates with BRAF status, we selected two established (Sbcl1, ME4405) and one patient-derived (Mel2622) cell lines belonging to the BRAF *wt* group, and three established (A375, LOX IMVI, ME4686) and one patient-derived (Mel2648) cell lines showing BRAF mutation.

### DT2216 potentiates the sensitivity of BRAF *wild type* melanoma cells to MEK or Mcl-1 inhibitors

**Fig. 3:**
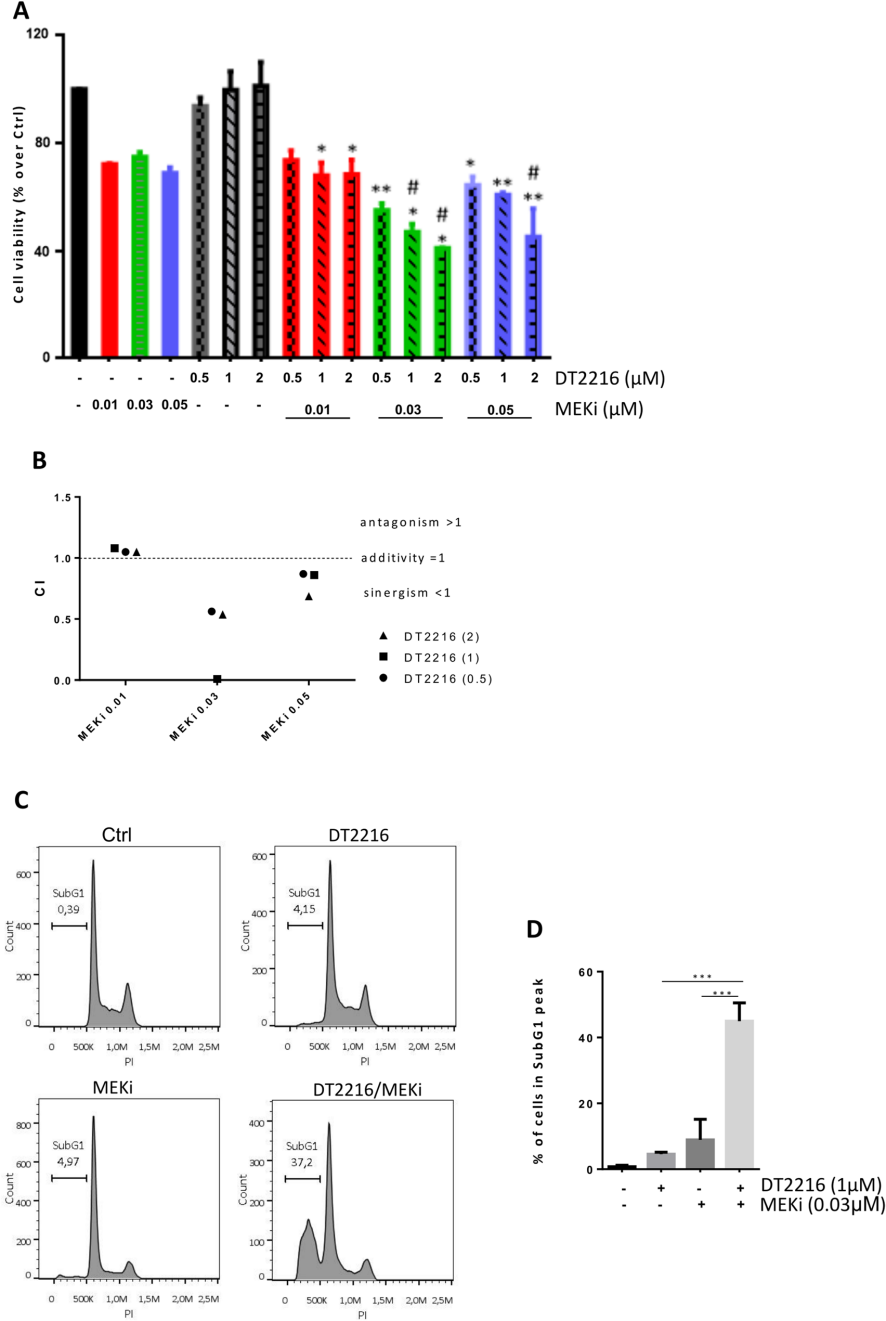
DT2216 potentiates the efficacy of Trametinib (MEKi) in BRAF *wild type* Sbcl1 melanoma cells. **A** Analysis of cell viability of cells treated with DT2216 (0.5-2µM) and MEKi (0.01–0.05µM), alone or in combination for 48 h. *p*-values were calculated between DT2216 and combination treatment *p<0.05, **p <0.01 and between MEKi and combination treatments #*p* <0.5. **B** Combination Index (CI) of cells treated as shown in (A). CI < 1 synergistic effect, CI = 1 additive effect, CI > 1 antagonistic effect. **C** Representative images of cytofluorimetric analysis of cell distribution in the cell cycle after treatment with 1µM DT2216 and 0.03µM MEKi, alone or in combination for 48 h. Percentage of cells in the subG1 peak, indicative of apoptosis, is reported. A, C Ctrl, control cells. **D** Quantification of cells in the subG1 peak, reported as mean ± SD of three independent experiments. *p*-values were calculated between single and combination treatments, ***p < 0.001

**Fig. 4:**
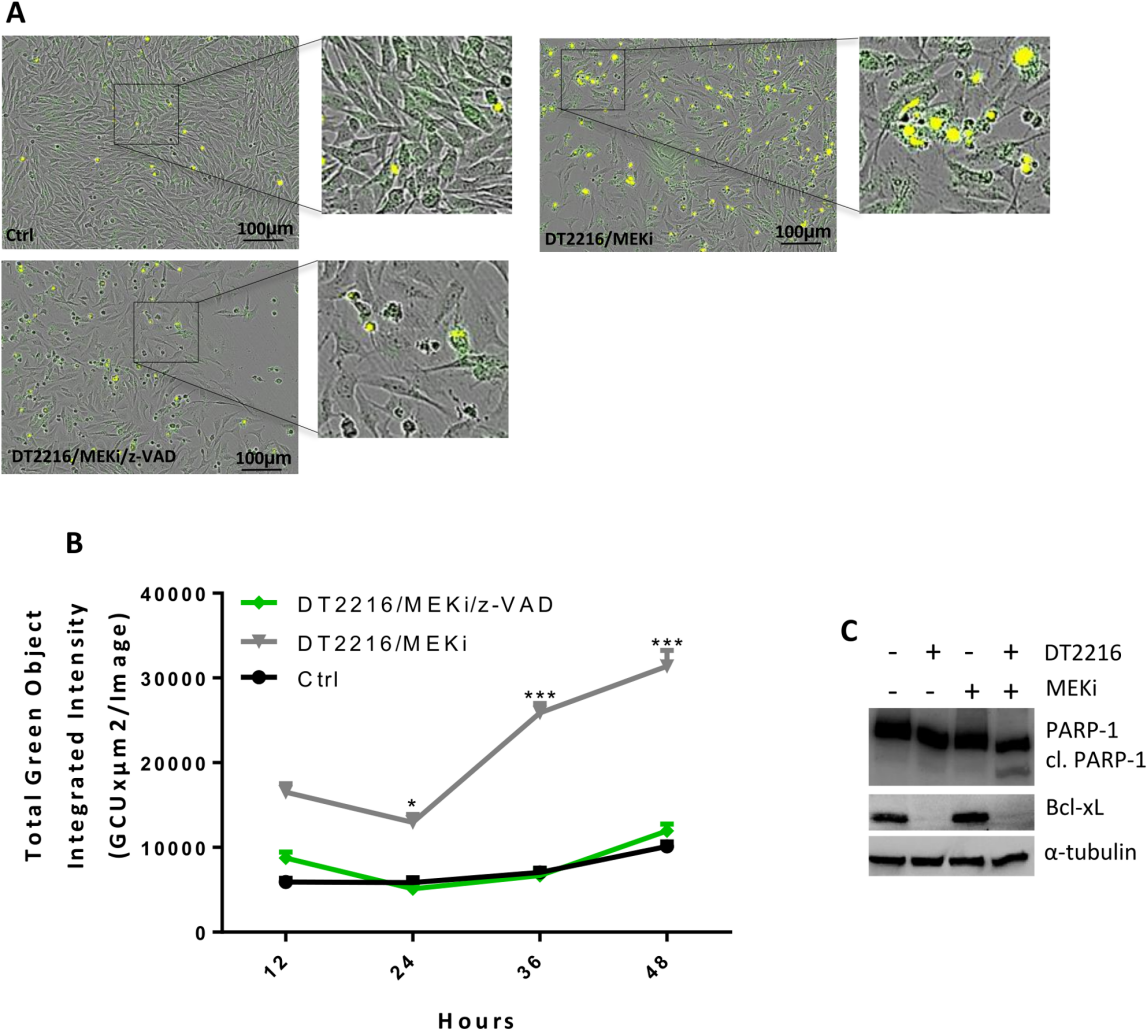
DT2216 potentiates the efficacy of Trametinib (MEKi) in BRAF *wild type* Sbcl1 melanoma cells. **A** Representative images of caspase 3/7 activation by live-cell imaging in cells treated with 1µM DT2216 and 0.03µM MEKi, in the absence or presence of 50µM z-VAD for 48 h. In yellow is represented the caspase activation signal. **B** Quantification of caspase 3/7 activation at the indicated time points, in cells treated as reported in (A). *p*-values were calculated comparing DT2216/MEKi combination treatment in the presence or absence of z-VAD, *p < 0.05, ***p < 0.001. A, B Ctrl, control cells. **C** Western blot analysis of Bcl-xL, PARP-1, and cleaved PARP-1 (cl. PARP-1) protein levels in cells treated with 1µM DT2216 and 0.03µM MEKi alone or in combination for 48 h. Western blot images are representative of two independent experiments with similar results. α-tubulin is shown as loading and transferring control

**Fig. 5:**
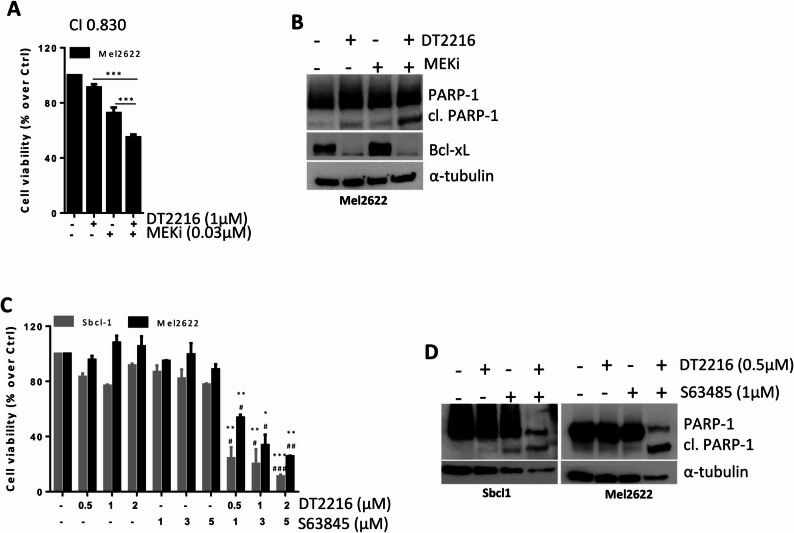
DT2216 potentiates the efficacy of Trametinib (MEKi) and S63845 (Mcl-1 inhibitor) in BRAF *wild type* Sbcl1 and Mel2622 melanoma cells. **A** Analysis of cell viability of Mel2622 cells treated with 1µM DT2216 and 0.03µM MEKi alone or in combination for 48 h. CI indicates Combination Index. **B** Western blot analysis of Bcl-xL, PARP-1, and cleaved PARP-1 (cl. PARP-1) protein levels in Mel2622 cells treated as in (A). **C** Analysis of cell viability of Sbcl1 and Mel2622 cells treated with increasing concentrations of DT2216 (0.5-2µM) and S63845 (1–5µM), alone or in combination for 48 h. **D** Western blot analysis of PARP-1 and cleaved PARP-1 (cl. PARP-1) protein levels in Sbcl1 and Mel2622 cells treated with 0.5µM DT2216 and 1µM S63845, alone or in combination for 48 h. A, C Results are reported as “viability of treated cells/viability of control cells (Ctrl)” × 100. *p*-values were calculated: between DT2216 and combination treatment ***p < 0.001 and between MEKi and combination treatment ***p < 0.001 (A); between DT2216 and combination treatments, *p < 0.05, **p < 0.01, ***p < 0.001 and S63845 and combination treatments #*p* < 0.05, ##*p *< 0.01 and ###*p* < 0.001 (C). B, D Western blot images are representative of two independent experiments with similar results. α-tubulin is shown as loading and transferring control

To analyse whether DT2216 treatment could improve the sensitivity of BRAF *wt* melanoma cells to target therapy, we firstly evaluated cell viability and induction of apoptosis exposing Sbcl1 cells to DT2216 (0.5-2µM) or MEKi (Trametinib 0.01–0.05µM) alone or in combination for 48 h. As shown in Fig. 3, the combinations DT2216/Trametinib at the two higher concentrations significantly reduced Sbcl1 cell viability (Fig. 3A), with a combination index < 1 indicative of a synergistic effect (Fig. 3B). Moreover, treating cells with 1µM DT2216 and 0.03µM Trametinib alone or in combination for 48 h, we observed an increase of the percentage of cells in the subG1 peak of cell cycle, in the combination regimen compared to the single treatments (Fig. 3C, D). To confirm induction of apoptosis, we analysed the activation of caspase 3/7 after exposure of Sbcl1 cells to 1µM DT2216 and 0.03µM Trametinib in combination for up to 48 h in the absence or presence of the caspase inhibitor z-VAD (50µM). As shown in Fig. 4A, B, the activated caspase 3/7 levels were near zero in control cells, while in the combination regimen the amount of caspase 3/7 levels was significantly higher starting from 24 h of treatment. Cells treated with the combination DT2216/Trametinib in presence of z-VAD showed caspase 3/7 levels similar to those of control cells, confirming apoptotic cell death. Cleavage of PARP-1 protein was observed by western blot analysis in the combined treatment (Fig. 4C). Similar results were obtained in the BRAF *wt* Mel2622 patient-derived melanoma cells, in terms of cell viability reduction (Fig. 5A) and apoptosis activation (Fig. 5B).

We next evaluated the effect of DT2216 on the expression of Bcl-2 and Mcl-1, two anti-apoptotic proteins involved in melanoma resistance to therapy [36]. As reported in Fig. S3, a significant increase of both proteins was observed after treatment of Sbcl1 and Mel2622 (data not shown) cells with DT2216. Considering these results, we investigated the effect of DT2216 in combination with the Bcl-2 specific inhibitor ABT-199/Venetoclax, used for the treatment of several hematologic malignancies [37], or with the Mcl-1 specific inhibitor S63845 [38]. To this aim, Sbcl1 and Mel2622 cell lines were treated with increasing concentrations of ABT-199 (1–5µM) or S63845 (1–5µM) for 48 h, alone or in combination with DT2216 (0.5-2µM). While a significant decrease in cell viability of both cell lines was observed only when the highest concentration of DT2216 (2µM) was combined with the highest concentration of ABT-199 (5µM) (Fig. S4A), all tested combinations DT2216 (0.5-2µM)/S63845 (1–5µM) induced a significant inhibition of cell viability (Fig. 5C). As reported in Fig. 5D, the combination of the lowest concentrations of DT2216 (0.5µM) and S63845 (1µM) induced cleavage of PARP-1 in both Sbcl1 and Mel2622 cells. Similar results were observed in the BRAF *wt* melanoma cell line, Me4405, not expressing Bcl-2 protein (Fig. S4B-D).

### DT2216 potentiates the effect of target therapy in BRAF mutated melanoma cells

**Fig. 6:**
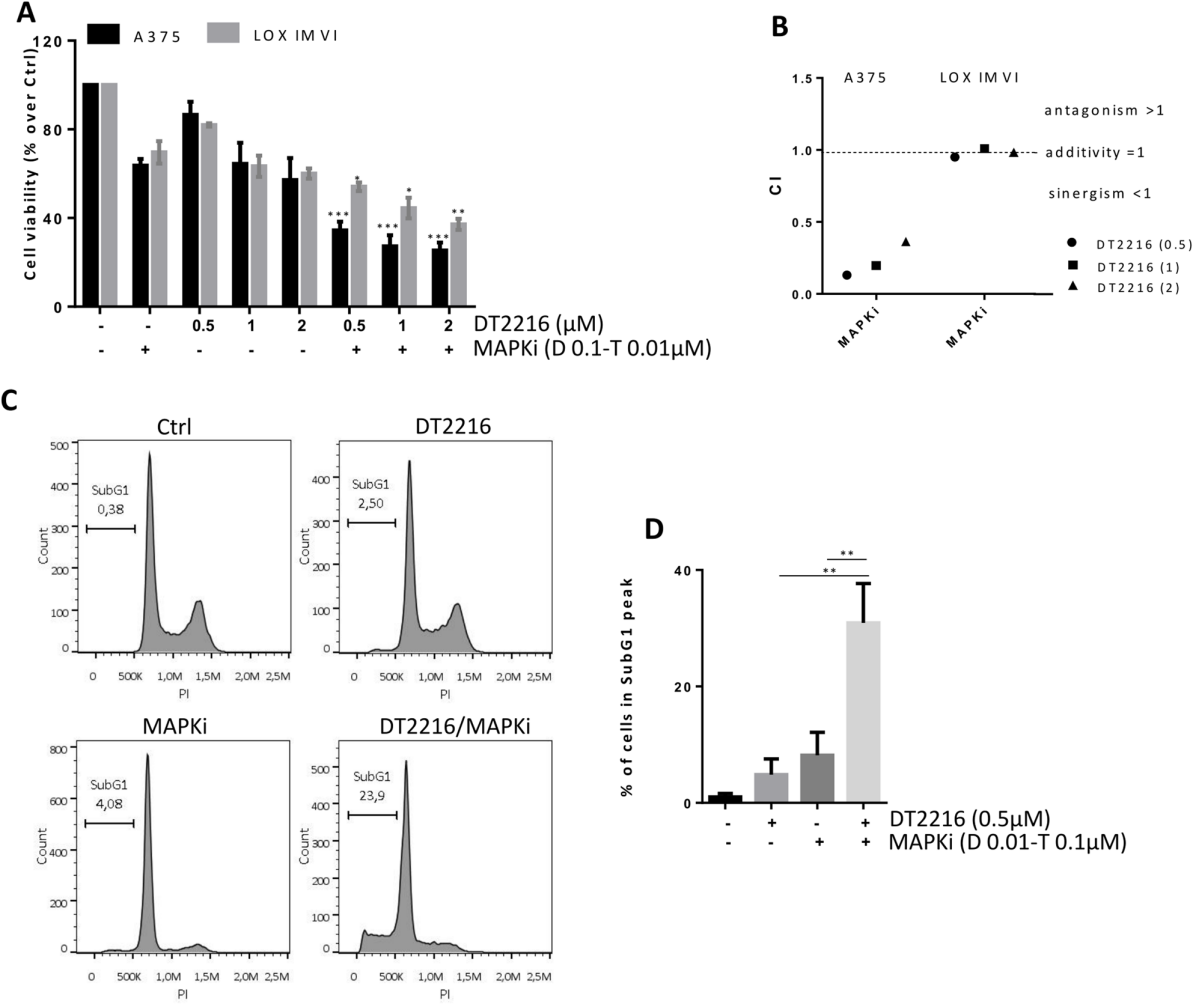
DT2216 potentiates the efficacy of MAPKi (Dabrafenib and Trametinib) in A375 and LOX IMVI BRAF mutated melanoma cells. **A** Analysis of cell viability of A375 and LOX IMVI cells treated with a fixed concentration of MAPKi (0.1µM Dabrafenib [D] and 0.01µM Trametinib [T]) and increasing concentration of DT2216 (0.5-2µM), alone or in combination for 48 h. *p*-values were calculated between DT2216 and combination treatment *p <0.05, **p <0.01, ***p < 0.001. **B** Combination Index (CI) of A375 and LOX IMVI cells treated as shown in (A). CI < 1 synergistic effect, CI = 1 additive effect, CI > 1 antagonistic effect. **C** Representative images of cytofluorimetric analysis of A375 cells distribution in cell cycle after treatment with 0.5µM DT2216 and MAPKi (0.1µM Dabrafenib and 0.01µM Trametinib), alone or in combination for 48 h. Percentage of cells in the subG1 peak, indicative of apoptosis, is reported. A, C Ctrl, control cells. **D** Quantification of cells in the subG1 peak, reported as mean ± SD of three independent experiments, *p*-values were calculated between single and combination treatments, **p < 0.01

**Fig. 7:**
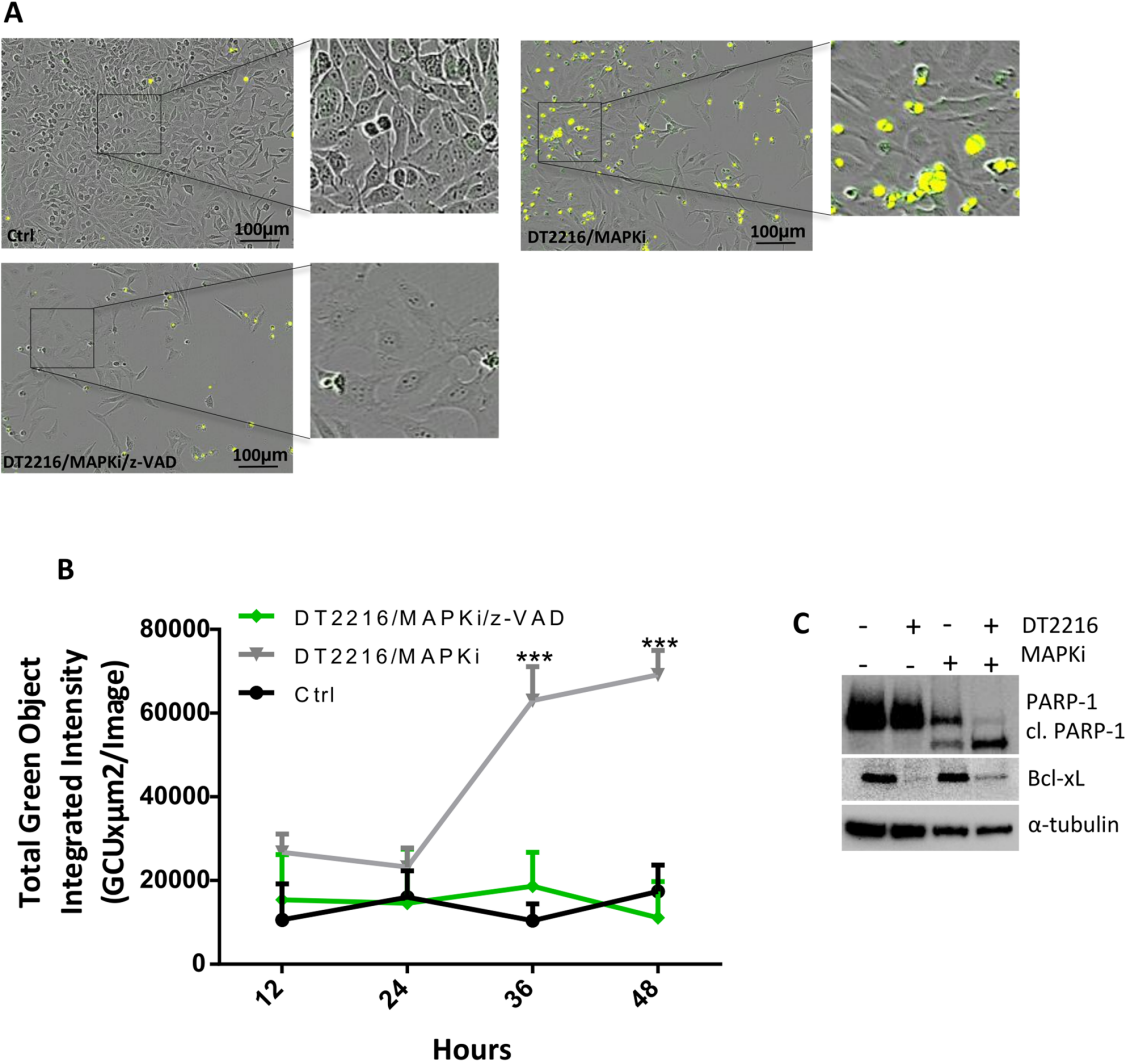
DT2216 potentiates the efficacy of MAPKi (Dabrafenib and Trametinib) in A375 BRAF mutated melanoma cells. **A** Representative images of caspase 3/7 activation by live-cell imaging in cells treated for 48 h with 0.5µM DT2216 and MAPKi (0.1µM Dabrafenib and 0.01µM Trametinib), in the absence or presence of 50µM z-VAD. In yellow is represented the caspase activation signal. **B** Quantification of caspase 3/7 activation at the indicated time points, in cells treated as reported in (A). *p*-values were calculated comparing DT2216/MAPKi combination treatment in the presence or absence of z-VAD, ***p < 0.001. A, B Ctrl, control cells. **C** Western blot analysis of Bcl-xL, PARP-1, and cleaved PARP-1 (cl. PARP-1) protein levels in cells treated with 0.5µM DT2216 and MAPKi (Dabrafenib 0.1µM and Trametinib 0.01µM), alone or in combination for 48 h. Western blot images are representative of two independent experiments with similar results. α- tubulin is shown as loading and transferring control

**Fig. 8:**
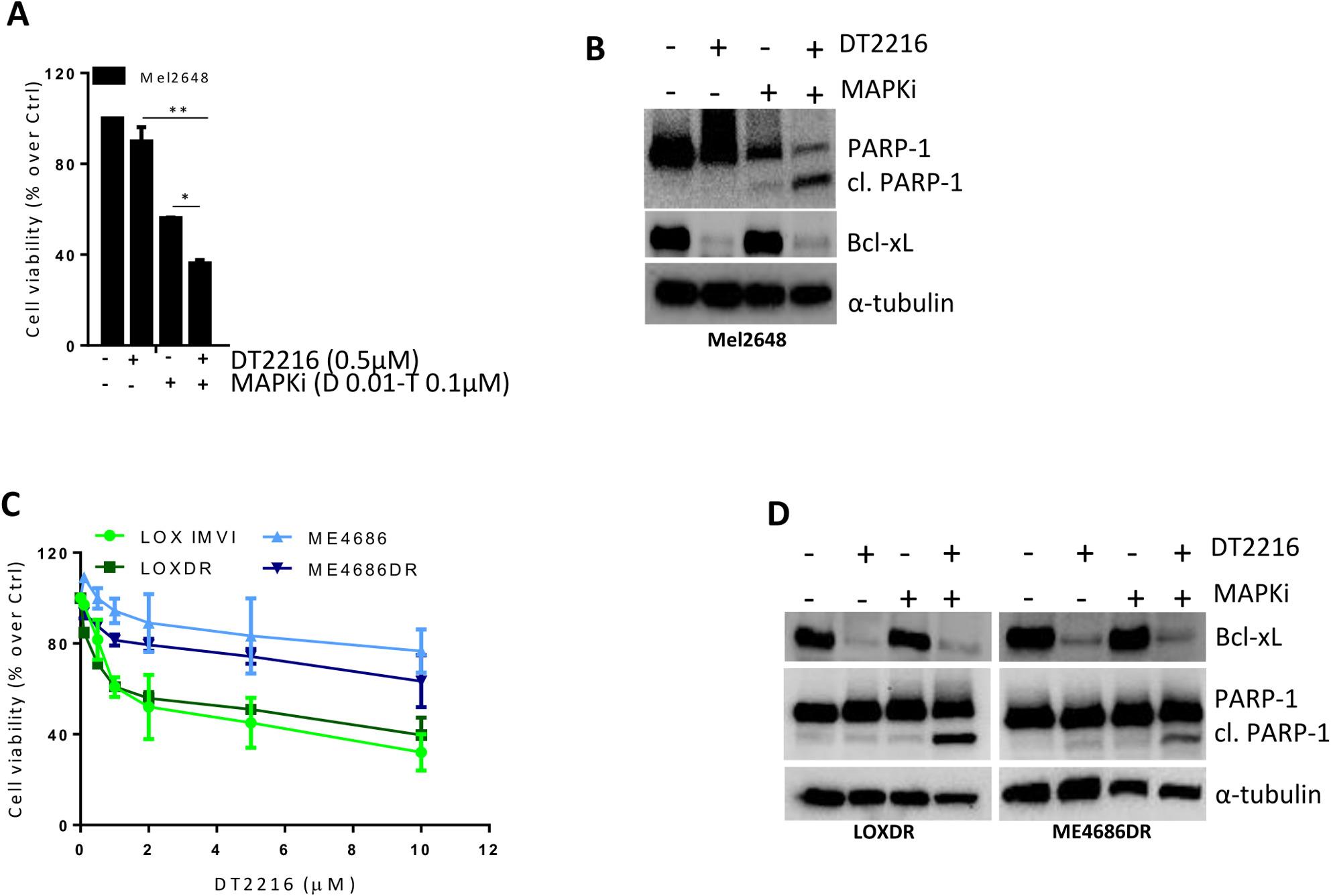
DT2216 potentiates the effect of MAPKi (Dabrafenib and Trametinib) in BRAF mutated Mel2648 and MAPKi resistant melanoma cells. **A** Analysis of cell viability of Mel2648 cells treated with 0.5µM DT2216 and MAPKi (0.1µM Dabrafenib [D] and 0.01µM Trametinib [T]), alone or in combination for 48 h. *p*-values were calculated between both single and combination treatments, *p < 0.5, **p < 0.01. **B** Western blot analysis of Bcl-xL, PARP-1 and cleaved PARP-1 (cl. PARP-1) protein levels in cells treated as reported in (A). **C** Analysis of cell viability of LOXDR and ME4686DR cells resistant to target therapy and their relative parental/sensitive cells treated with concentrations of DT2216 ranging from 0.1µM to 10µM for 72 h. Results are reported as “viability of treated cells/viability of control cells (Ctrl)” × 100 and as mean ± SD of three independent experiments. **D** Western blot analysis of Bcl-xL, PARP-1 and cleaved PARP-1 (cl. PARP-1) protein levels in LOXDR and ME4686DR cells treated with 0.5µM DT2216 and MAPKi (0.3µM Dabrafenib and 0.03µM Trametinib) alone or in combination for 48 h. B, D Western blot images are representative of two independent experiments with similar results. α- tubulin is shown as loading and transferring control

By using BRAF^V600E^ mutated melanoma cell lines (A375 and LOX IMVI), we investigated if DT2216 could also potentiate the efficacy of BRAFi + MEKi (MAPKi), a target therapy that, together with immunotherapy, represents the standard treatment for melanoma patients carrying BRAF mutations [39]. To this aim, A375 and LOX IMVI cells were treated with a fixed concentration of MAPKi (0.1µM Dabrafenib/0.01µM Trametinib) and increasing concentration of DT2216 (0.5-2µM) alone or in combination for 48 h. As reported in Fig. 6A, in both cell lines, all combinations induced a significant reduction in cell viability when compared to both DT2216 or Dabrafenib/Trametinib single treatments, with a synergistic effect for A375 (CI < 1) and an additive effect for LOX IMVI (CI = 1) (Fig. 6B). Moreover, in both cell lines we observed a significant increase of percentage of cells in the subG1 peak of cell cycle (Fig. 6C, D, Fig. S5A, B), when the regimen DT2216/MAPKi was compared to the other treatments. Activation of caspase 3/7 was also measured in A375 cells treated for up to 48 h with 0.5µM DT2216 and MAPKi (0.1µM Dabrafenib/0.01µM Trametinib), in the absence or presence of 50µM z-VAD. As shown in Fig. 7A, B, caspase 3/7 levels were near zero in control cells, while in the combination regimen the amount of caspase 3/7 levels was significantly higher starting from 36 h. Cells treated with the combination DT2216/MAPKi in presence of z-VAD, showed caspase 3/7 levels similar to control cells, confirming apoptotic cell death. Western blot analysis evidenced an increased cleavage of PARP-1 protein when the combined treatment was compared to single ones (Fig. 7C). Similar results were observed in LOX IMVI cell line, where the activation of caspase 3/7 decreased after exposure to DT2216 and MAPKi in the presence of z-VAD (Fig. S5C). An increase of cleaved PARP-1 after the combination treatment was also evidenced (Fig. S5D). The ability of DT2216 to significantly potentiate the efficacy of MAPKi in terms of reduction of cell viability and induction of apoptosis was also confirmed in BRAF mutated Mel2648 patient-derived (Fig. 8A, B) and ME4686 established (Fig. S6A-C) melanoma cells.

We also investigated the effect of DT2216 in two melanoma cell lines resistant to BRAFi/MEKi, LOXDR and ME4686DR and relative parental/sensitive cells (Fig. S7). As reported in Fig. 8C, 72 h exposure to DT2216 at concentrations ranging from 0.1µM to 10µM reduced cell viability in both parental and resistant cells. Moreover, PARP-1 cleavage was evident when resistant cells were exposed to DT2216/MAPKi combined treatment for 48 h compared to single treatments (Fig. 8D**).**

### DT2216 sensitizes BRAF mutant melanoma cells to MAPKi In vivo

**Fig. 9:**
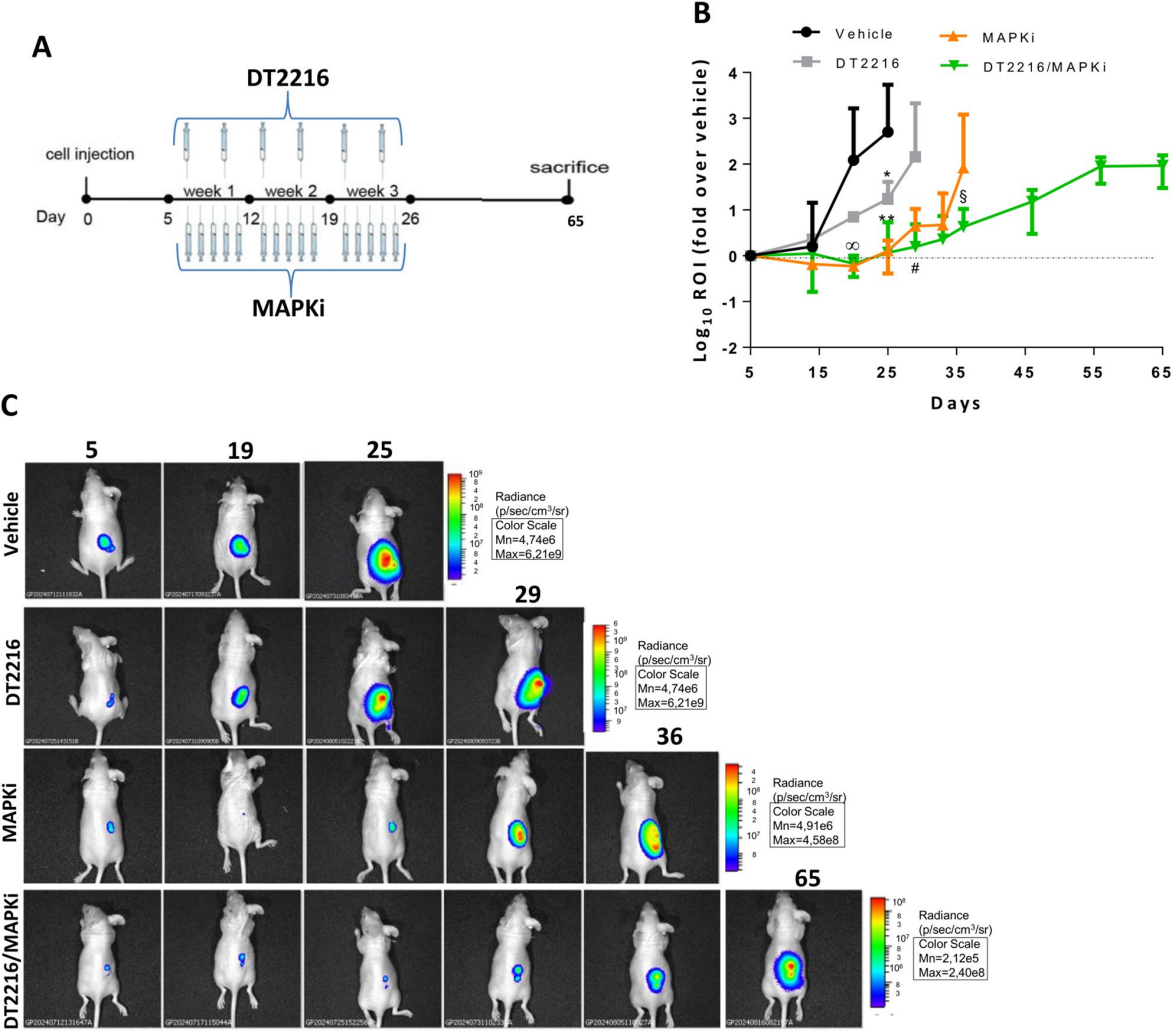
DT2216 potentiates the efficacy of MAPKi (Dabrafenib and Trametinib) in A375-derived xenograft mice. **A** Schematic timeline of in vivo experiments in nude mice. **B** Analysis of tumor growth after A375luc cells injection and treatment with vehicle, DT2216 (15mg/Kg), MAPKi (5mg/kg Dabrafenib and 0.01mg/kg Trametinib) or DT2216/MAPKi for three weeks. Analysis of tumor growth is expressed as ROI. *p*-values were calculated between vehicle and MAPKi or DT2216/MAPKi combination (∞ *p* < 0.05), between vehicle and DT2216 (*p < 0.05), vehicle and MAPKi or DT2216/MAPKi combination (**p < 0.01), between DT2216 and combination treatments (#*p *< 0.05), or between MAPKi and the combination treatments (§*p* < 0.05). **C** Representative images of in vivo growth of A375 xenografts at the indicated days (5–65). Experiments were repeated twice

Based on the in vitro results, we investigated the antitumoral activity of the different treatments in in vivo models. To this aim, mice carrying A375luc melanoma were treated with DT2216 or MAPKi (Dabrafenib + Trametinib) alone or in combination (DT2216/MAPKi) for three weeks, as reported in Fig. 9A. Tumors were monitored through both manual calliper (tumor volume) (Fig. S8A) and imaging analysis (ROI) (Fig. 9B, C). As expected, and as we previously demonstrated [34], an initial tumor shrinkage from day 5 to day 25 was observed in mice treated with MAPKi when compared to vehicle and DT2216 treated mice. A similar trend was also evidenced in the animals treated with the DT2216/MAPKi combination regimen. Starting from day 25, tumors exposed to MAPKi and DT2216/MAPKi started to regrow, with a slower tumor growth in the DT2216/MAPKi group compared to the MAPKi group. Due to reaching the maximum tolerated weight, the mice treated with DT2216 or MAPKi were sacrificed, respectively, at days 29 and 36 after the beginning of the treatments. Interestingly, the DT2216/MAPKi combination group showed a longer disease control, being sacrificed 30 days after the MAPKi group (at day 65) (Fig. 9B, Fig. S8A). Notably, all treatments were highly tolerated and no significant changes in diet consumption, postural/behavioral habits, and body weight, were observed (Fig. S8B).

## Discussion

High levels of Bcl-xL anti-apoptotic protein correlate with tumor progression, resistance to chemotherapy and poor prognosis in tumors from different origins, including melanoma. For these reasons, the relevance of using molecules able to inhibit or degrade this protein as a new therapeutic approach for cancer therapy [5, 8, 40–42] is increasingly studied. Several Bcl-xL inhibitors have been developed and tested in clinical trials alone or in combinatorial regimes. On-target platelet toxicity, frequently experienced in patients with both hematological [43] and solid [44, 45] malignancies, limited clinical application of Navitoclax. Even though dose escalation phase I/II study (NCT02079740) performed on patients with advanced solid malignancies, including melanoma, treated with Navitoclax in combination with Trametinib, demonstrated a reduced grade of thrombocytopenia [46], the identification of Bcl-xL inhibiting/degrading agents with low platelets toxicity represents a medical need. As VHL is poorly expressed in human platelets [29], the proteasome-dependent Bcl-xL specific PROTAC degrader, DT2216, does not affect platelets viability, thus overcoming the problem of thrombocytopenia [26]. For this reason, DT2216 has been proposed as a new strategy to achieve safe and potent antitumor activity [29].

In the present study, we investigated the sensitivity to DT2216 of melanoma models. We also evaluated whether DT2216 can increase the efficacy of target therapy that, together with the immunotherapy, represents the first-line treatment for advanced melanoma, although with a limited efficacy in a large fraction of melanoma patients [2, 3]. To this aim, in vitro established and patient-derived BRAF *wt* and mutated melanoma cells, and in vivo melanoma models were used.

Our findings demonstrate that DT2216 induced specific and persistent degradation of Bcl-xL protein regardless of BRAF status in human melanoma cells, all expressing detectable levels of Bcl-xL protein. The use of the proteasome inhibitor, MG132, confirmed that Bcl-xL protein degradation by DT2216 involves the ubiquitin-proteasome system. Moreover, DT2216 reduced cell viability of melanoma cells in a concentration-dependent manner, even if at different extent. Of note, a positive correlation between Bcl-xL protein degradation and viability reduction was observed, being the cells with higher degradation of Bcl-xL those more sensitive to DT2216. In agreement with published findings [47], our data reveal the existence of a cell line-specific sensitivity to DT2216, without any correlation with the expression levels of the VHL E3 ligase in the cells, thus indicating the relevance of other mechanisms in the response of melanoma cells to DT2216. Moreover, as PROTACs efficacy has been reported to correlate with the kinetic of assembly and dissociation of ternary complex [48], the different sensitivity could be related to the different stability of ternary complex. Notably, the response to DT2216 is independently from BRAF status, showing two BRAF *wt* cell lines, Sbcl1 and Mel2622, low and high values of both IC_50_ and DC_50_, respectively. This information could be useful to understand which patients could benefit from DT2216 treatment.

Of note, DT2216 resulted not to being toxic to human immortalized fibroblasts and endothelial cells. Needs to be emphasized that the Me4405 melanoma cell line, in which Bcl-2 protein was not detectable [34], showed decreased viability after exposure to DT2216, thus excluding the relevance of Bcl-2 protein in the sensitivity to DT2216 of melanoma cells. In several melanoma cell lines DT2216 treatment even induced an increase of Bcl-2 protein.

Even thoughin vitro and in vivo studies demonstrated the potentiating effect of DT2216 in combinatorial regimes both in hematological [25] and solid tumors, including lung [26, 49], hepatocellular [27], colon [28] and pancreatic [50] carcinoma, to the best of our knowledge, our findings put in evidence, for the first time, the ability of DT2216 to increase the efficacy of target therapy in melanoma. DT2216 activity was effective regardless BRAF status and, more importantly, was evidenced in melanoma cells resistant to BRAF/MEK inhibition treatment. When combined with target therapy, DT2216 induced a potentiating effect both in BRAF *wt* and mutated melanoma cells, in terms of reduction of cell viability, with synergistic or additive interactions. Activation of the apoptotic process, evaluated as percentage of cells in the subG1 peak of the cell cycle, activation of caspase 3/7 and expression/cleavage of PARP-1 protein, was observed after the combinatorial treatments. Apoptotic cell death was confirmed by using the caspase inhibitor, z-VAD. Interestingly, combined exposure to DT2216/MAPKi was more effective than single treatments also in melanoma cells resistant to target therapy. As some melanoma cells belonging to the same group (i.e. A375 and LOX IMVI both with a V600E mutation in the BRAF protein) show similar expression levels of Bcl-xL protein, and similar response to DT2216 in terms of Bcl-xL degradation and reduction of cell viability, we exclude that differences in the combination index (synergistic or additive) are due to the response to DT2216 single treatment or to the BRAF status. We could hypothesize that other genetic or phenotypic features can contribute to this difference.

The antitumoral efficacy of DT2216 in combinatorial regimes was also confirmed in melanoma xenograft models. Our in vivo results carried out in mice models evidenced that combining DT2216 with targeted therapy, the standard of care for melanoma patients, significantly reduced tumor growth and conferred a longer disease control. All these data indicate that (i) the potentiated effect of DT2216 with molecular target therapy is independent of BRAF mutational state of melanoma cells; (ii) the reduction of high basal levels of Bcl-xL could decrease the apoptotic threshold in melanoma cells that cannot be overcome by target therapy as single treatment, that represents a limitation of BRAF mutated melanoma, and (iii) the Bcl-xL inhibition is also effective in melanoma cells resistant to BRAF/MEK inhibitors.

Of note, in agreement with data reported in literature, in which the use of BH3 mimetics [5, 38, 51–54] induced an increase of anti-apoptotic proteins of Bcl-2 family, we found that the treatment with DT2216 determined a rise of Bcl-2 and/or Mcl-1 protein expression in several melanoma cell lines, thus exerting a sort of compensatory mechanism. This evidence prompted us to combine DT2216 with the Bcl-2 specific inhibitor ABT-199, currently used for the treatment of several haematological malignancies [37], or the Mcl-1 specific inhibitor S63845, effective and tolerable in various cancer models [38]. While only high concentrations of DT2216/ABT-199 reduced cell viability, the combined treatment DT2216/S63845, induced cell death at all tested concentrations. These results, in agreement with previously published papers [26, 55], demonstrate the efficacy of PROTAC-based approach to degrade Bcl-xL protein in combination with Mcl-1 inhibitors for the therapy of solid tumors. This strategy could show the advantage of reducing the on-target platelet and cardiomyocytic toxicity observed after treatment with, Bcl-xL [22, 54, 56] or Mcl-1 [57] inhibitors, respectively. Thereby, overcoming the damage frequently due to the combination of Bcl-xL and Mcl-1 inhibitors. We are aware that the use of alternative approaches to selectively depleting Mcl-1 protein in tumor cells without triggering cardiotoxicity, or to indirectly target Mcl-1, as reported for anti-mitotic and DNA replication or HER2-targeting drugs [55, 58], would have greater translational implications.

## Conclusion

Our findings support the importance of DT2216 for the treatment of some tumors in the near future, and in particular to improve outcomes of patients with reduced therapeutic opportunities or who do not respond to first-line therapy. Moreover, our results could rise the development of a dual PROTAC targeting both Bcl-xL and Mcl-1, in order not only to treat tumors which depend on these proteins, but also to overcome a possible resistance to therapy induced by the increase of Mcl-1 after treatment.

Preclinical studies support the future perspective on the use of DT2216 in clinical practice, especially when combined with drugs already submitted for approval by the FDA. A phase I clinical trial, with no available results, was completed in November 2023 to study the toxicity, pharmacokinetics and efficacy of this PROTAC (NCT04886622) on patients with advanced or metastatic solid tumors not responsive to approved therapies. A phase I/II recruiting trial (NCT06620302) testing the safety, side effects and best concentration of DT2216 in combination regimen is ongoing in patients with solid tumors that has relapsed or that has not responded to previous treatment. Moreover, a phase Ib dose-escalation study (NCT06964009) is going to recruit patients to establish the recommended phase II dose and to evaluate the safety of combined dosing of DT2216 with paclitaxel in recurrent platinum-resistant ovarian cancer.

Aware that further studies are needed to improve PROTACs specificity/delivery/solubility and to reduce off-tissue effects, these data highlighted the opportunity to give new therapeutical options to patients with metastatic melanoma who are not responsive to standard therapy, offering a new drug with low levels of platelets toxicity. Patients with both BRAF *wt* and mutated advanced melanoma, as well as patients with cancer types relying on Bcl-xL for their survival, could benefit from therapies including DT2216.

## Supplementary Information

Supplementary Material 1.

Supplementary Material 2.

## Data Availability

All data and materials used are available within the manuscript.

## Abbreviations

A375luc: A375 cells expressing luciferase
AJCC: American Joint Committee on Cancer
LOXDR: LOX IMVI double resistant cells
MAPKi: Mitogen-Activated Protein Kinase inhibitor
MEKi: Mitogen-Activated Protein Kinase Kinase inhibitor
ME4686DR: ME4686 double resistant cells
POI: Protein of interest
PROTAC: Proteolysis Targeting Chimera
VHL: Von Hippel-Lindau
z-VAD: z-VAD-fmk

## References

[1] Kennedy LB, Salama AKS. A marathon not a sprint: improving outcomes for patients with metastatic melanoma in 2022 and beyond. JCO Oncol Pract. 2022. 10.1200/OP.22.00012. 35196070 10.1200/OP.22.00012

[2] Siegel RL, Miller KD, Wagle NS, Jemal A. Cancer statistics, 2023. CA Cancer J Clin. 2023. 10.3322/caac.21763. 36633525 10.3322/caac.21763

[3] Fateeva A, Eddy K, Chen S. Current state of melanoma therapy and next steps: battling therapeutic resistance. Cancers (Basel). 2024. 10.3390/cancers16081571. 38672652 10.3390/cancers16081571 PMC11049326

[4] Luciano AM, Perez-Oliva AB, Mulero V, Del Bufalo D. Bcl-xL: A focus on melanoma pathobiology. Int J Mol Sci. 2021. 10.3390/ijms22052777. 33803452 10.3390/ijms22052777 PMC7967179

[5] Kaloni D, Diepstraten ST, Strasser A, Kelly GL. BCL-2 protein family: attractive targets for cancer therapy. Apoptosis. 2023. 10.1007/s10495-022-01780-7. 36342579 10.1007/s10495-022-01780-7 PMC9950219

[6] Scherr A, Gdynia G, Salou M, Radhakrishnan P, Duglova K, Heller A, et al. Bcl-xL is an oncogenic driver in colorectal cancer. Cell Death Dis. 2016. 10.1038/cddis.2016.233. 27537525 10.1038/cddis.2016.233 PMC5108319

[7] Sela Y, Li J, Maheswaran S, Norgard R, Yuan S, Hubbi M, et al. Bcl-xL enforces a Slow-Cycling state necessary for survival in the Nutrient-Deprived microenvironment of pancreatic cancer. Cancer Res. 2022. 10.1158/0008-5472.CAN-22-0431. 35315913 10.1158/0008-5472.CAN-22-0431 PMC9117449

[8] Ikezawa K, Hikita H, Shigekawa M, Iwahashi K, Eguchi H, Sakamori R, et al. Increased Bcl-xL expression in pancreatic neoplasia promotes carcinogenesis by inhibiting senescence and apoptosis. Cell Mol Gastroenterol Hepatol. 2017. 10.1016/j.jcmgh.2017.02.001. 28948203 10.1016/j.jcmgh.2017.02.001 PMC5604117

[9] Alcon C, Gomez Tejeda Zanudo J, Albert R, Wagle N, Scaltriti M, Letai A, et al. ER + Breast cancer strongly depends on MCL-1 and BCL-xL Anti-Apoptotic proteins. Cells. 2021. 10.3390/cells10071659. 34359829 10.3390/cells10071659 PMC8304651

[10] Bodac A, Mayet A, Rana S, Pascual J, Bowler AD, Roh V, et al. Bcl-xL targeting eliminates ageing tumor-promoting neutrophils and inhibits lung tumor growth. EMBO Mol Med. 2024. 10.1038/s44321-023-00013-x. 38177532 10.1038/s44321-023-00013-x PMC10897164

[11] Castellanet O, Ahmad F, Vinik Y, Mills GB, Habermann B, Borg J, et al. BCL-XL blockage in TNBC models confers vulnerability to Inhibition of specific cell cycle regulators. Theranostics. 2021. 10.7150/thno.60503. 34646365 10.7150/thno.60503 PMC8490507

[12] Giorgini S, Trisciuoglio D, Gabellini C, Desideri M, Castellini L, Colarossi C, et al. Modulation of bcl-xL in tumor cells regulates angiogenesis through CXCL8 expression. Mol Cancer Res. 2007. 10.1158/1541-7786.MCR-07-0088. 17699103 10.1158/1541-7786.MCR-07-0088

[13] Gabellini C, Gomez-Abenza E, Ibanez-Molero S, Tupone MG, Perez-Oliva AB, de Oliveira S, et al. Interleukin 8 mediates bcl-xL-induced enhancement of human melanoma cell dissemination and angiogenesis in a zebrafish xenograft model. Int J Cancer. 2018. 10.1002/ijc.31075. 28949016 10.1002/ijc.31075

[14] Luciano AM, Di Martile M, Perez-Oliva AB, Di Caprio M, Foddai ML, Buglioni S, et al. Exploring association of melanoma-specific Bcl-xL with tumor immune microenvironment. J Exp Clin Cancer Res. 2023. 10.1186/s13046-023-02735-9. 37488586 10.1186/s13046-023-02735-9 PMC10364435

[15] Alcon C, Kovatcheva M, Morales-Sanchez P, Lopez-Polo V, Torres T, Puig S, et al. HRK downregulation and augmented BCL-xL binding to BAK confer apoptotic protection to therapy-induced senescent melanoma cells. Cell Death Differ. 2024. 10.1038/s41418-024-01417-z. 39627361 10.1038/s41418-024-01417-z PMC11982230

[16] Wang L, Doherty GA, Judd AS, Tao Z, Hansen TM, Frey RR, et al. Discovery of A-1331852, a First-in-Class, Potent, and Orally-Bioavailable BCL-X(L) inhibitor. ACS Med Chem Lett. 2020. 10.1021/acsmedchemlett.9b00568. 33062160 10.1021/acsmedchemlett.9b00568 PMC7549103

[17] Tao Z, Hasvold L, Wang L, Wang X, Petros AM, Park CH, et al. Discovery of a potent and selective BCL-XL inhibitor with in vivo activity. ACS Med Chem Lett. 2014. 10.1021/ml5001867. 25313317 10.1021/ml5001867 PMC4190639

[18] Lessene G, Czabotar PE, Sleebs BE, Zobel K, Lowes KN, Adams JM, et al. Structure-guided design of a selective BCL-X(L) inhibitor. Nat Chem Biol. 2013. 10.1038/nchembio.1246. 23603658 10.1038/nchembio.1246

[19] Tse C, Shoemaker AR, Adickes J, Anderson MG, Chen J, Jin S, et al. ABT-263: a potent and orally bioavailable Bcl-2 family inhibitor. Cancer Res. 2008. 10.1158/0008-5472.CAN-07-5836. 18451170 10.1158/0008-5472.CAN-07-5836

[20] Schoenwaelder SM, Jarman KE, Gardiner EE, Hua M, Qiao J, White MJ, et al. Bcl-xL-inhibitory BH3 mimetics can induce a transient thrombocytopathy that undermines the hemostatic function of platelets. Blood. 2011. 10.1182/blood-2011-04-347849. 21673344 10.1182/blood-2011-04-347849

[21] Lv D, Pal P, Liu X, Jia Y, Thummuri D, Zhang P, et al. Development of a BCL-xL and BCL-2 dual degrader with improved anti-leukemic activity. Nat Commun. 2021. 10.1038/s41467-021-27210-x. 34824248 10.1038/s41467-021-27210-x PMC8617031

[22] Kolb R, De U, Khan S, Luo Y, Kim M, Yu H, et al. Proteolysis-targeting chimera against BCL-X(L) destroys tumor-infiltrating regulatory T cells. Nat Commun. 2021. 10.1038/s41467-021-21573-x. 33627663 10.1038/s41467-021-21573-x PMC7904819

[23] Cornu M, Lemaitre T, Kieffer C, Voisin-Chiret AS. PROTAC 2.0: expanding the frontiers of targeted protein degradation. Drug Discov Today. 2025. 10.1016/j.drudis.2025.104376. 40348076 10.1016/j.drudis.2025.104376

[24] Garber K. The PROTAC gold rush. Nat Biotechnol. 2022. 10.1038/s41587-021-01173-2. 34907403 10.1038/s41587-021-01173-2

[25] Gress V, Roussy M, Boulianne L, Bilodeau M, Cardin S, El-Hachem N, et al. CBFA2T3::GLIS2 pediatric acute megakaryoblastic leukemia is sensitive to BCL-XL Inhibition by navitoclax and DT2216. Blood Adv. 2024. 10.1182/bloodadvances.2022008899. 37729615 10.1182/bloodadvances.2022008899 PMC10787250

[26] Khan S, Kellish P, Connis N, Thummuri D, Wiegand J, Zhang P, et al. Co-targeting BCL-X(L) and MCL-1 with DT2216 and AZD8055 synergistically inhibit small-cell lung cancer growth without causing on-target toxicities in mice. Cell Death Discov. 2023. 10.1038/s41420-022-01296-8. 36588105 10.1038/s41420-022-01296-8 PMC9806104

[27] Shebl B, Ng D, Lalazar G, Rosemore C, Finkelstein TM, Migler RD, et al. Targeting BCL-XL in fibrolamellar hepatocellular carcinoma. JCI Insight. 2022. 10.1172/jci.insight.161820. 36073545 10.1172/jci.insight.161820 PMC9536265

[28] Jenkins LJ, Luk IY, Chionh F, Tan T, Needham K, Ayton J, et al. BCL-X(L) inhibitors enhance the apoptotic efficacy of BRAF inhibitors in BRAF(V600E) colorectal cancer. Cell Death Dis. 2024. 10.1038/s41419-024-06478-z. 38429301 10.1038/s41419-024-06478-z PMC10907349

[29] Khan S, Zhang X, Lv D, Zhang Q, He Y, Zhang P, et al. A selective BCL-X(L) PROTAC degrader achieves safe and potent antitumor activity. Nat Med. 2019. 10.1038/s41591-019-0668-z. 31792461 10.1038/s41591-019-0668-z PMC6898785

[30] Zhang X, Thummuri D, Liu X, Hu W, Zhang P, Khan S, et al. Discovery of PROTAC BCL-X(L) degraders as potent anticancer agents with low on-target platelet toxicity. Eur J Med Chem. 2020. 10.1016/j.ejmech.2020.112186. 32145645 10.1016/j.ejmech.2020.112186 PMC7433031

[31] Mahadevan D, Barve M, Mahalingam D, Parekh J, Kurman M, Strauss J, et al. First in human phase 1 study of DT2216, a selective BCL-xL degrader, in patients with relapsed/refractory solid malignancies. J Hematol Oncol. 2025. 10.1186/s13045-025-01753-8. 41225624 10.1186/s13045-025-01753-8 PMC12613848

[32] Valentini E, D’Aguanno S, Di Martile M, Montesano C, Ferraresi V, Patsilinakos A, et al. Targeting the anti-apoptotic Bcl-2 family proteins: machine learning virtual screening and biological evaluation of new small molecules. Theranostics. 2022. 10.7150/thno.64233. 35265218 10.7150/thno.64233 PMC8899577

[33] Di Martile M, Farini V, Consonni FM, Trisciuoglio D, Desideri M, Valentini E, et al. Melanoma-specific bcl-2 promotes a protumoral M2-like phenotype by tumor-associated macrophages. J Immunother Cancer. 2020. 10.1136/jitc-2019-000489. 32269145 10.1136/jitc-2019-000489 PMC7254128

[34] Valentini E, Di Martile M, Brignone M, Di Caprio M, Manni I, Chiappa M, et al. Bcl-2 family inhibitors sensitize human cancer models to therapy. Cell Death Dis. 2023. 10.1038/s41419-023-05963-1. 37460459 10.1038/s41419-023-05963-1 PMC10352371

[35] Wang Z, He N, Guo Z, Niu C, Song T, Guo Y, et al. Proteolysis targeting chimeras for the selective degradation of Mcl-1/Bcl-2 derived from nonselective target binding ligands. J Med Chem. 2019. 10.1021/acs.jmedchem.9b00919. 31389699 10.1021/acs.jmedchem.9b00919

[36] Lee EF, Harris TJ, Tran S, Evangelista M, Arulananda S, John T, et al. BCL-XL and MCL-1 are the key BCL-2 family proteins in melanoma cell survival. Cell Death Dis. 2019. 10.1038/s41419-019-1568-3. 31019203 10.1038/s41419-019-1568-3 PMC6482196

[37] Lasica M, Anderson MA. Review of venetoclax in CLL, AML and multiple myeloma. J Pers Med. 2021. 10.3390/jpm11060463. 34073976 10.3390/jpm11060463 PMC8225137

[38] Kotschy A, Szlavik Z, Murray J, Davidson J, Maragno AL, Le Toumelin-Braizat G, et al. The MCL1 inhibitor S63845 is tolerable and effective in diverse cancer models. Nature. 2016. 10.1038/nature19830. 27760111 10.1038/nature19830

[39] Natarelli N, Aleman SJ, Mark IM, Tran JT, Kwak S, Botto E, et al. A review of current and pipeline drugs for treatment of melanoma. Pharmaceuticals (Basel). 2024. 10.3390/ph17020214. 38399429 10.3390/ph17020214 PMC10892880

[40] Trisciuoglio D, Del Bufalo D. New insights into the roles of antiapoptotic members of the Bcl-2 family in melanoma progression and therapy. Drug Discov Today. 2021. 10.1016/j.drudis.2021.01.027. 33545382 10.1016/j.drudis.2021.01.027

[41] Ramesh P, Medema JP. BCL-2 family deregulation in colorectal cancer: potential for BH3 mimetics in therapy. Apoptosis. 2020. 10.1007/s10495-020-01601-9. 32335811 10.1007/s10495-020-01601-9 PMC7244464

[42] Kawiak A, Kostecka A. Regulation of Bcl-2 family proteins in Estrogen Receptor-Positive breast cancer and their implications in endocrine therapy. Cancers (Basel). 2022. 10.3390/cancers14020279. 35053443 10.3390/cancers14020279 PMC8773933

[43] Roberts AW, Seymour JF, Brown JR, Wierda WG, Kipps TJ, Khaw SL, et al. Substantial susceptibility of chronic lymphocytic leukemia to BCL2 inhibition: results of a phase I study of navitoclax in patients with relapsed or refractory disease. J Clin Oncol. 2012. 10.1200/JCO.2011.34.7898. 22184378 10.1200/JCO.2011.34.7898 PMC4979082

[44] Rudin CM, Hann CL, Garon EB, Ribeiro de Oliveira M, Bonomi PD, Camidge DR, et al. Phase II study of single-agent navitoclax (ABT-263) and biomarker correlates in patients with relapsed small cell lung cancer. Clin Cancer Res. 2012. 10.1158/1078-0432.CCR-11-3090. 22496272 10.1158/1078-0432.CCR-11-3090 PMC3715059

[45] Gandhi L, Camidge DR, Ribeiro de Oliveira M, Bonomi P, Gandara D, Khaira D, et al. Phase I study of navitoclax (ABT-263), a novel Bcl-2 family inhibitor, in patients with small-cell lung cancer and other solid tumors. J Clin Oncol. 2011. 10.1200/JCO.2010.31.6208. 21282543 10.1200/JCO.2010.31.6208 PMC4668282

[46] Corcoran RB, Do KT, Kim JE, Cleary JM, Parikh AR, Yeku OO, et al. Phase I/II study of combined BCL-xL and MEK Inhibition with navitoclax and Trametinib in KRAS or NRAS mutant advanced solid tumors. Clin Cancer Res. 2024. 10.1158/1078-0432.CCR-23-3135. 38456660 10.1158/1078-0432.CCR-23-3135 PMC11061595

[47] Dolle A, Adhikari B, Kramer A, Weckesser J, Berner N, Berger L, et al. Design, Synthesis, and evaluation of WD-Repeat-Containing protein 5 (WDR5) degraders. J Med Chem. 2021. 10.1021/acs.jmedchem.1c00146. 33980013 10.1021/acs.jmedchem.1c00146

[48] Schwalm MP, Kramer A, Dolle A, Weckesser J, Yu X, Jin J, et al. Tracking the PROTAC degradation pathway in living cells highlights the importance of ternary complex measurement for PROTAC optimization. Cell Chem Biol. 2023. 10.1016/j.chembiol.2023.06.002. 37354907 10.1016/j.chembiol.2023.06.002

[49] Zhang W, Jin Y, Wang J, Gu M, Wang Y, Zhang X, et al. Co-delivery of PROTAC and SiRNA via novel liposomes for the treatment of malignant tumors. J Colloid Interface Sci. 2025. 10.1016/j.jcis.2024.08.185. 39222609 10.1016/j.jcis.2024.08.185

[50] Han S, Tushoski-Aleman GW, Zhang P, Zheng G, Zhou D, Huo Z, et al. A novel regimen for pancreatic ductal adenocarcinoma targeting MEK, BCL-xL, and EGFR. Neoplasia. 2025. 10.1016/j.neo.2024.101070. 39541736 10.1016/j.neo.2024.101070 PMC11609319

[51] Lee Y, Wang L, Huang C, Shi Y, Chang L. ABT-263-induced MCL1 upregulation depends on autophagy-mediated 4EBP1 downregulation in human leukemia cells. Cancer Lett. 2018. 10.1016/j.canlet.2018.06.019. 29913235 10.1016/j.canlet.2018.06.019

[52] Hatok J, Racay P. Bcl-2 family proteins: master regulators of cell survival. Biomol Concepts. 2016. 10.1515/bmc-2016-0015. 27505095 10.1515/bmc-2016-0015

[53] Tantawy SI, Sarkar A, Hubner S, Tan Z, Wierda WG, Eldeib A, et al. Mechanisms of MCL-1 protein stability induced by MCL-1 antagonists in B-Cell malignancies. Clin Cancer Res. 2023. 10.1158/1078-0432.CCR-22-2088. 36346691 10.1158/1078-0432.CCR-22-2088 PMC9852224

[54] Zhang X, Tao Y, Xu Z, Jiang B, Yang X, Huang T, et al. Sorafenib and SIAIS361034, a novel PROTAC degrader of BCL-x(L), display synergistic antitumor effects on hepatocellular carcinoma with minimal hepatotoxicity. Biochem Pharmacol. 2024. 10.1016/j.bcp.2024.116542. 39284500 10.1016/j.bcp.2024.116542

[55] Thummuri D, Khan S, Underwood PW, Zhang P, Wiegand J, Zhang X, et al. Overcoming gemcitabine resistance in pancreatic cancer using the BCL-X(L)-Specific degrader DT2216. Mol Cancer Ther. 2022. 10.1158/1535-7163.MCT-21-0474. 34667112 10.1158/1535-7163.MCT-21-0474 PMC8742767

[56] He Y, Koch R, Budamagunta V, Zhang P, Zhang X, Khan S, et al. DT2216-a Bcl-xL-specific degrader is highly active against Bcl-xL-dependent T cell lymphomas. J Hematol Oncol. 2020. 10.1186/s13045-020-00928-9. 32677976 10.1186/s13045-020-00928-9 PMC7364785

[57] Thomas RL, Roberts DJ, Kubli DA, Lee Y, Quinsay MN, Owens JB, et al. Loss of MCL-1 leads to impaired autophagy and rapid development of heart failure. Genes Dev. 2013. 10.1101/gad.215871.113. 23788623 10.1101/gad.215871.113 PMC3701192

[58] Zhang L, Wei Y, Luo M, Ren S, Zhan X, Wang C, et al. Both direct and indirect suppression of MCL1 synergizes with BCLXL Inhibition in preclinical models of gastric cancer. Cell Death Dis. 2025. 10.1038/s41419-025-07481-8. 40075071 10.1038/s41419-025-07481-8 PMC11904182

