# Supplementary material for: PROTAC-mediated degradation of Bcl-xL potentiates target therapy in preclinical melanoma models
Source: J Exp Clin Cancer Res. 2026 Jan 8;45:37. doi: 10.1186/s13046-025-03635-w (PMC12870073; doi:10.1186/s13046-025-03635-w)
Supplement: Supplementary file 1 — Supplementary Material 1. [file 13046_2025_3635_MOESM1_ESM.pdf]

Table S1. Molecular features of Mel2622 and Mel2648 patient-derived melanoma cells

	**Immunophenotype**		**Next Generation Sequencing analysis**			**OncoKB/ESCAT levels**	**Note**
**Cell line**	**MART1**	**S100**	**Gene**	**VAF* (%)**	**AA **Change**	3A/IIIB	
**Mel2622**	+	+	NRAS	99.85	p.Q61L		polymorphism
			KIT	47.55	p.M541L		polymorphism
			ABL1	99.50	p.K247R		polymorphism
			TP53	49.65	p.P72R	1/IA	
**Mel2648**	+	+	BRAF	54.62	p.V600E		polymorphism
			TP53	58.65	p.P72R		polymorphism
			STK11	48.92	p.P326T		

Table S2. Clinicopathological and molecular characteristics of melanoma samples

**Patient FFPE**	**Diagnosis**	**Breslow Thickness**	**AJCC Classification**	**Gene**	**VAF* (%)**	**AA** Change**	**OncoKB/ESCAT levels**	**Note**
**NM246_23 (Mel2622)**	Superficial Spreading Melanoma	2,2 mm	pT4a	NRAS	54.30	p.Q61L	3A/IIIB	
				KIT	42.23	p.M541L		polymorphism
				ABL1	70.62	p.K247R		polymorphism
				TP53	45.32	p.P72R		polymorphism
**NM132_22 (Mel2648)**	Polypoid Melanoma	9 mm	pT4b	BRAF	47.20	p.V600E	1/IA	
				TP53	50.62	p.P72R		polymorphism
				STK11	54.23	p.P326T		polymorphism

* Variant Allele Frequency

** Aminoacids

Fig.S1

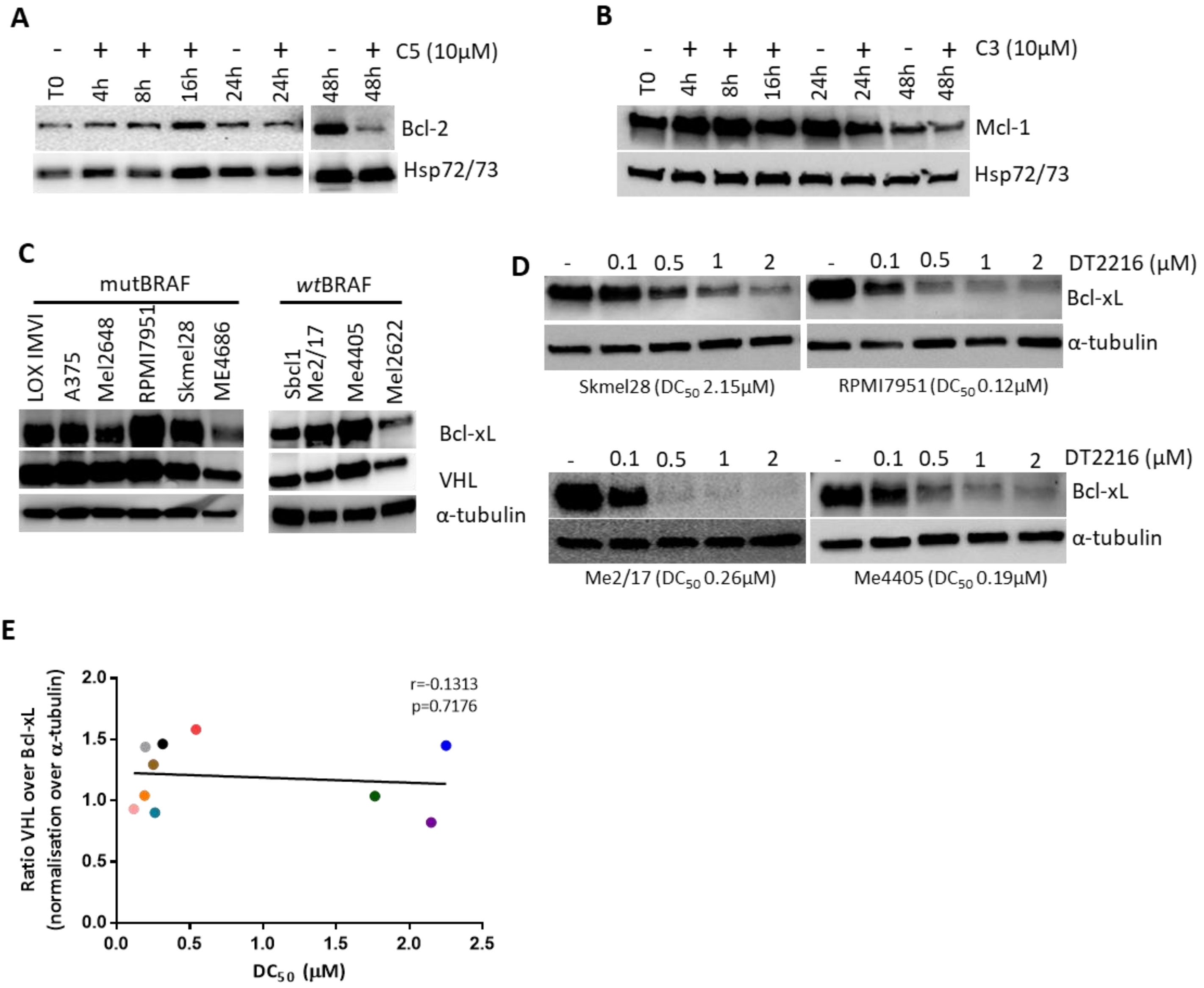

Fig. S1. C5, C3 and DT2216 PROTACs degrade, respectively, Bcl-2, Mcl-1 and Bcl-xL proteins, in melanoma cells. Western blot analysis of (A) Bcl-2 and (B) Mcl-1 protein expression in A375 cells after treatment with 10μM C5 or 10μM C3 for times ranging from 4h to 48h. (C) Western blot analysis of Bcl-xL and VHL protein expression in a panel of both BRAF mutated (mutBRAF) and *wild type* (*wt*BRAF) melanoma cells. (D) Western blot analysis of Bcl-xL protein expression after treatment with doses of DT2216 ranging from 0.1μM to 2μM for 24h. BRAF mutated (upper panel) and BRAF *wild type* (lower panel) melanoma cell lines were used. Numbers in brackets indicate DC_{50} values. (E) Correlation analysis between DC_{50} values and the ratio levels of VHL and Bcl-xL. (A-D) Western blot images are representative of two independent experiments with similar results. Hsp72/73 or α-tubulin are shown as loading and transferring control.

Fig.S2

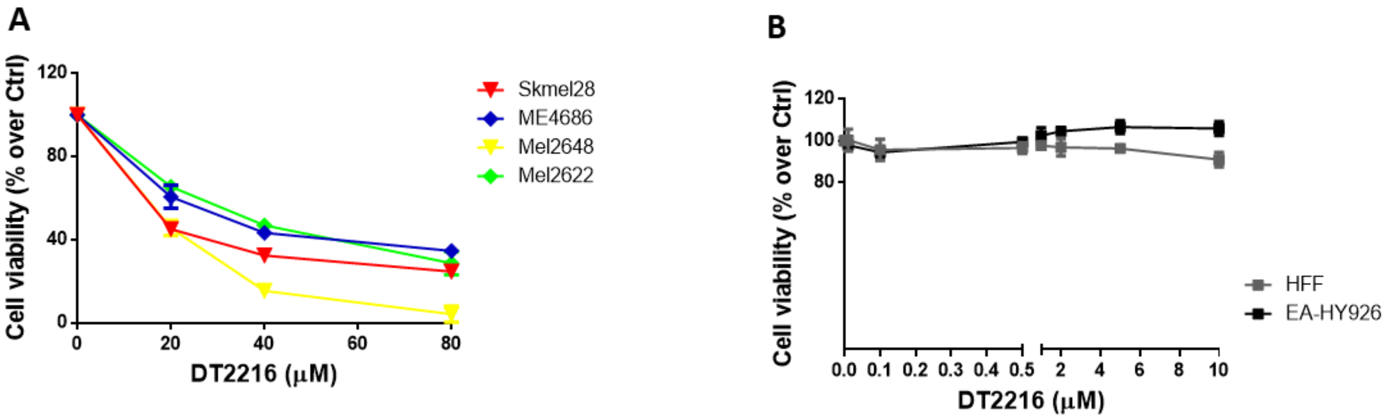

Fig. S2. DT2216 reduces cell viability of a panel of melanoma cells. (A) Analysis of cell viability of the indicated melanoma cell lines treated with concentrations of DT2216 ranging from 20μM to 80μM for 72h. (B) Analysis of cell viability of human fibroblasts (HFF) and endothelial cells (EA-HY926) treated with concentrations of DT2216 ranging from 0.1 to 10μM for 72h. (A,B) Results are reported as "viability of treated cells/viability of untreated control cells (Ctrl)" × 100, and as mean ± SD of three independent experiments.

Fig.S3

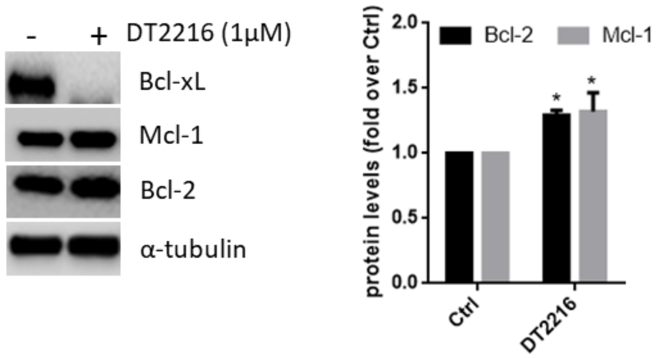

Fig. S3. DT2216 treatment increases Mcl-1 and Bcl-2 protein expression in BRAF *wild type* Sbcl1 melanoma cells. Western blot analysis of Bcl-xL, Mcl-1 and Bcl-2 protein levels in cells treated with 1μM DT2216 for 48h (left) and relative densitometric analysis of Bcl-2 and Mcl-1 protein levels (right). Western blot images are representative of two independent experiments with similar results. α-tubulin is shown as loading and transferring control.

Fig.S4

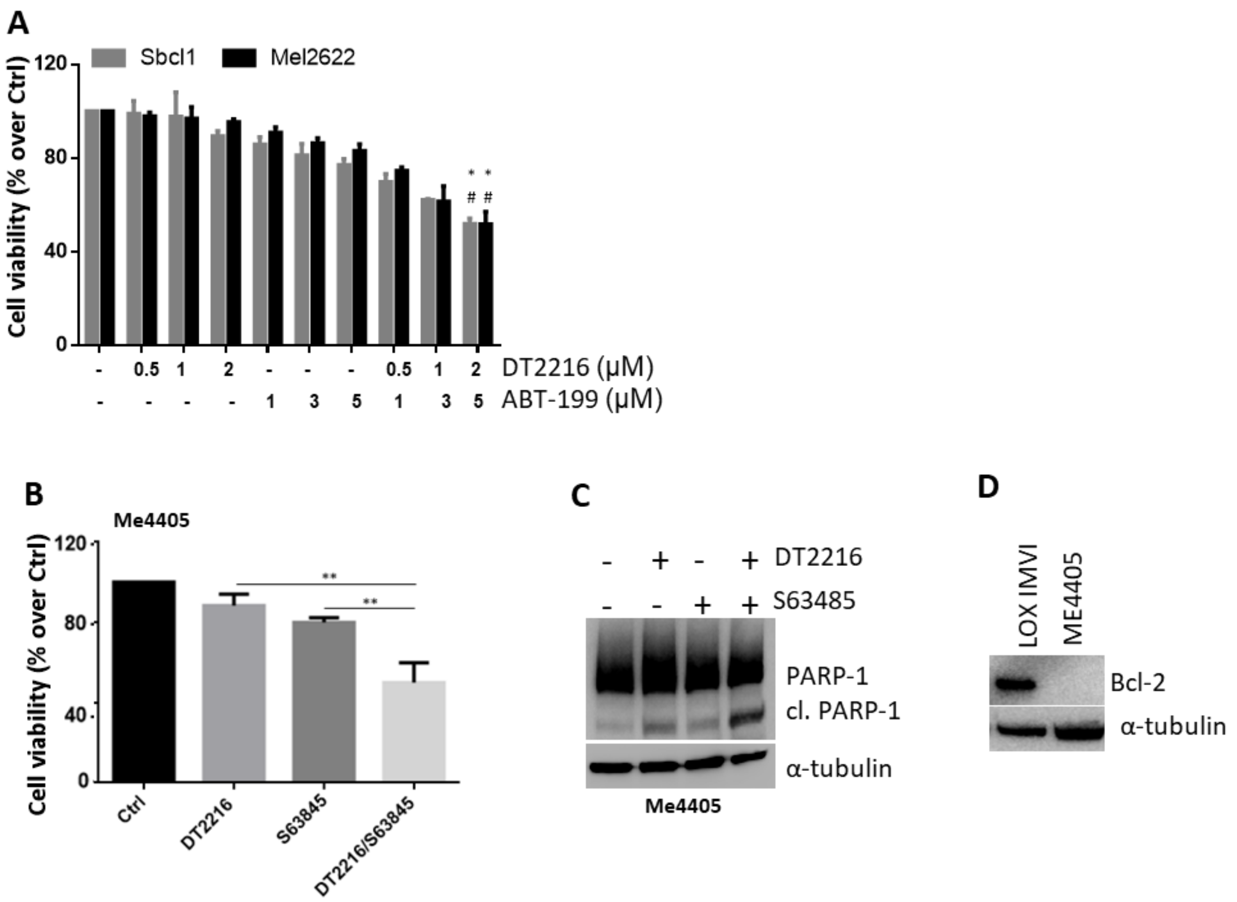

Fig. S4. DT2216 cooperates with ABT-199 and S63845 to reduce cell viability of BRAF *wild type* melanoma cells. (A) Analysis of cell viability of Sbcl1 and Mel2622 cells treated with DT2216 (0.5-2μM) and ABT-199 (1-5μM), alone or in combination for 48h. p-values were calculated between DT2216 and combination treatments (*p<0.05), or between ABT-199 and combination treatments ($^{\#}$p<0.05). (B) Cell viability and (C) western blot analysis of PARP-1 and cleaved PARP-1 (cl. PARP-1) protein levels in Me4405 cells treated with 0.5μM DT2216 and 1μM S63485, alone or in combination for 48h. (B) p-values were calculated between single and combination treatments, **p<0.001. (D) Western blot analysis of Bcl-2 protein levels in LOX IMVI and Me4405 cells. (C,D) Western blot images are representative of two independent experiments with similar results. α- tubulin is shown as loading and transferring control. (A,B) Results are reported as “viability of treated cells/viability of control cells (Ctrl)” × 100 and as mean ± SD of three independent experiments.

Fig.S5

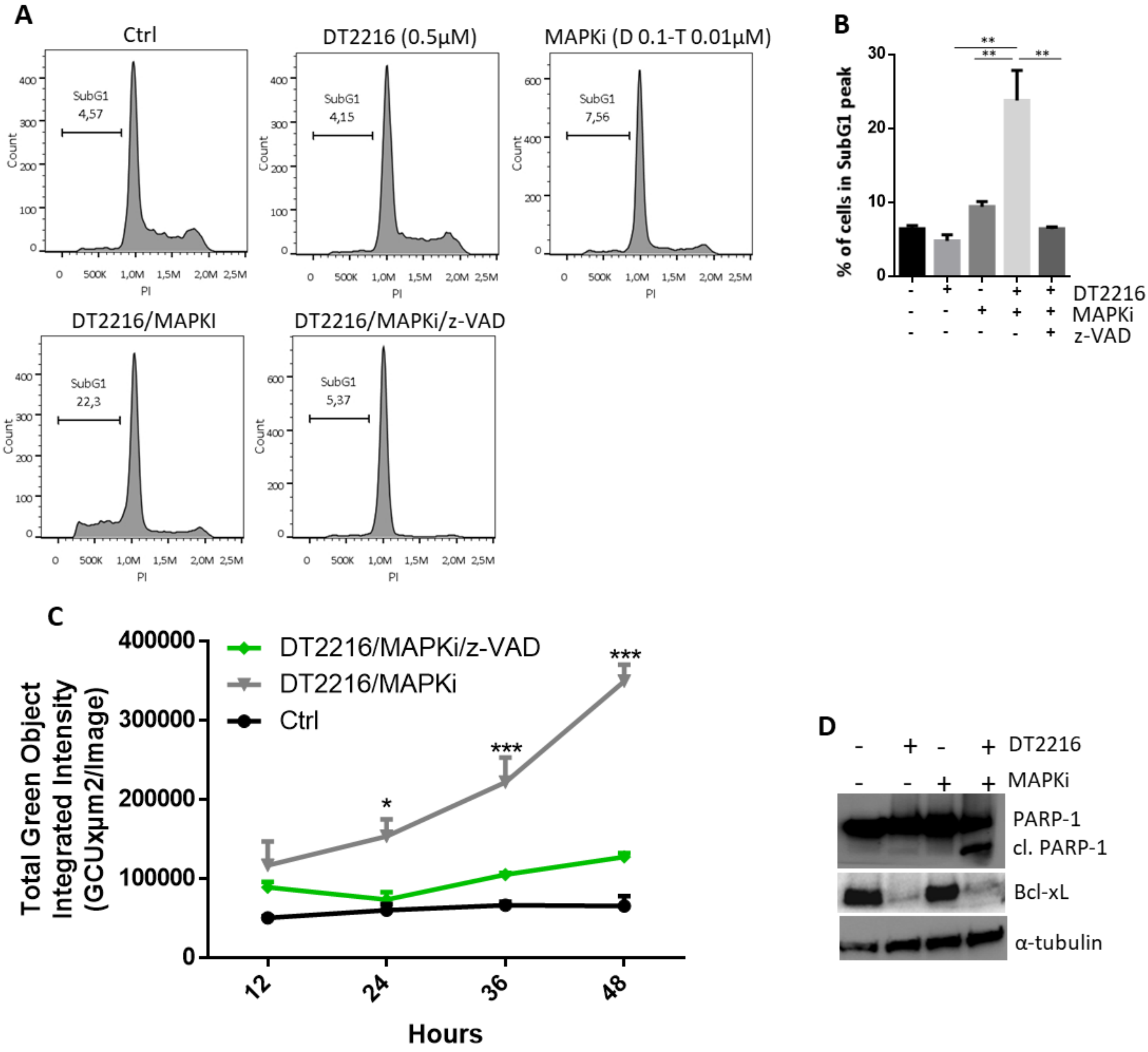

Fig. S5 DT2216 potentiates the effect of MAPKi (Dabrafenib and Trametinib), inducing apoptosis in LOX IMVI BRAF mutated melanoma cells. (A) Representative images of cytofluorimetric analysis of cells distribution in the different phases of cell cycle after treatment with 0.5µM DT2216 and MAPKi (0.1µM Dabrafenib [D] and 0.01µM Trametinib [T]), alone or in combination for 48h, in presence or absence of 50µM z-VAD. Percentage of cells in the subG1 peak is reported. (B) Quantification of cells in the subG1 peak reported as mean ± SD from three independent experiments after treatment as reported in (A). p values were calculated between single and

combination treatments and between combination regimen in the presence or absence of z-VAD **p<0.001. (C) Quantification analysis, performed at indicated time points, of caspase 3/7 activation in LOX IMVI cells treated as in (A). p-values were calculated between DT2216/MAPKi combination and the treatment in the presence of 50µM z-VAD, *p<0.01, ***p<0.0001. (D) Western blot analysis of Bcl-xL, PARP-1, and cleaved PARP-1 (cl. PARP-1) protein levels in cells treated with 0.5µM DT2216 and MAPKi (0.1µM Dabrafenib [D] and 0.01µM Trametinib [T]), alone or in combination for 48h. Western blot images are representative of two independent experiments with similar results. α- tubulin is shown as loading and transferring control.

Fig.S6

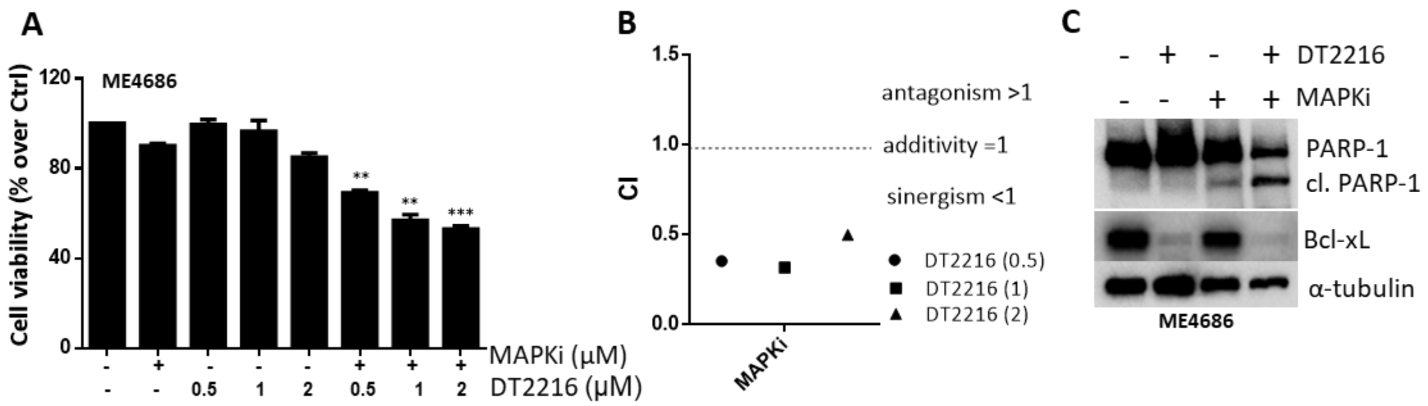

Fig. S6. DT2216 shows synergistic effect with MAPKi (Dabrafenib and Trametinib) in BRAF mutated ME4686 melanoma cells. (A) Analysis of cell viability of cells treated with a fixed concentration of MAPKi (0.1µM Dabrafenib [D] and 0.01µM Trametinib [T]) and increasing concentration of DT2216 (0.5-2µM), alone or in combination for 48h. Results are reported as “viability of treated cells/viability of control cells (Ctrl)” × 100 and as mean ± SD of three independent experiments. p-values were calculated between single and combination treatments, **p<0.001 and ***p<0.0001. (B) Combination Index (CI) of cells treated as in (A). CI<1 synergistic effect, CI=1 additive effect, CI>1 antagonistic effect. (C) Western blot analysis of Bcl-xL, PARP-1 and cleaved PARP-1 (cl. PARP-1) protein levels in cells treated with 0.5µM DT2216 and MAPKi (0.1µM Dabrafenib [D] and 0.01µM Trametinib [T]), alone or in combination for 48h. Western blot images are representative of two independent experiments with similar results. α-tubulin is shown as loading and transferring control.

Fig.S7

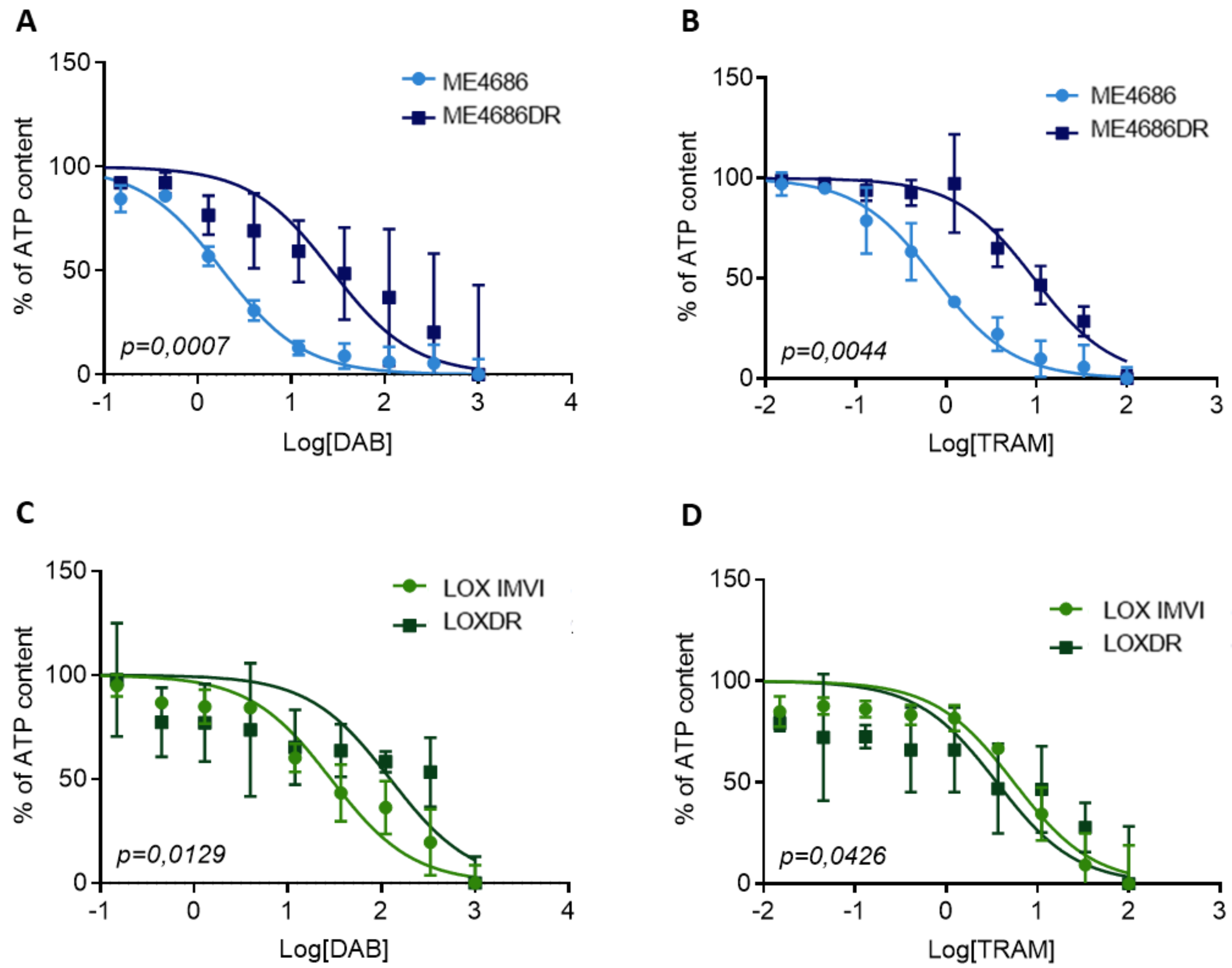

Fig. S7. Dose–response curves of sensitive and resistant melanoma cell lines treated with Dabrafenib (BRAFi) and Trametinib (MEKi). (A,B) Analysis of cell viability of ME4686 and ME4686DR and (C,D) LOX IMVI and LOXDR, exposed to different concentrations of (A,C) Dabrafenib starting at 1µM with nine 1:3 serial dilutions, and (B,D) Trametinib starting at 100nM with nine 1:3 serial dilutions. Cell viability was quantified using the CellTiter-Glo ATP-based luminescent assay. p-values were calculated between the treatments performed on sensitive and resistant cells.

Fig.S8

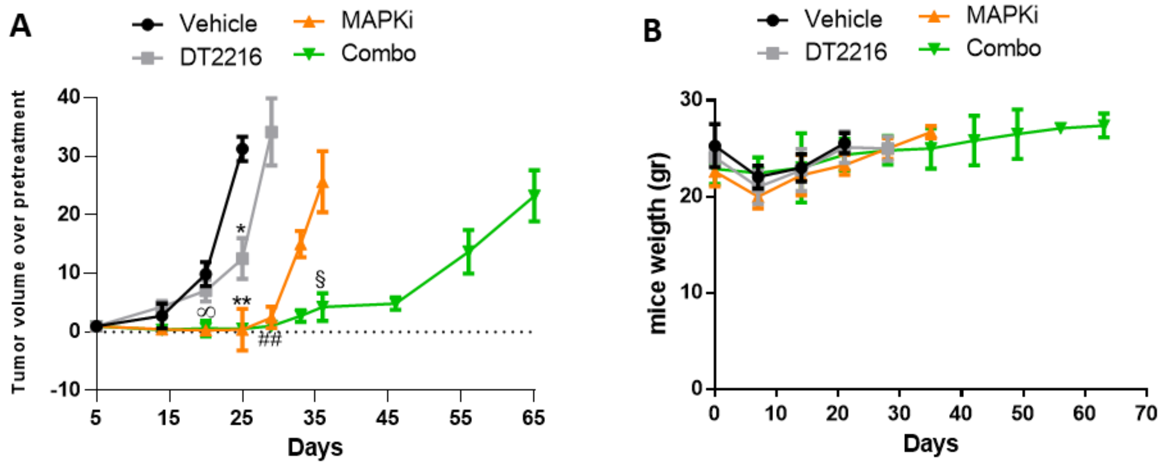

Fig. S8 DT2216 potentiates the effect of MAPKi (Dabrafenib and Trametinib) in A375-derived xenografts mice. (A) Analysis of tumor growth after A375luc cells injection and treatment with vehicle, DT2216 (15mg/Kg), MAPKi (5mg/kg Dabrafenib and 0.01mg/kg Trametinib) or DT2216/MAPKi for three weeks. Volumes have been shown after normalization over tumor size before starting treatments (pretreatment). p-values were calculated between vehicle and both MAPKi and combination DT2216/MAPKi treatments (∞ $p<0.05$), between vehicle and DT2216 (*$p<0.05$), vehicle and both MAPKi and DT2216/MAPKi (**$p<0.001$), between DT2216 and combination treatments (##$p<0.05$), or between MAPKi and the combination treatments (§$p<0.05$). (B) Analysis of mice weight during treatments described in (A). Experiments were repeated twice.
