# Supplementary material for: PROTAC-mediated degradation of Bcl-xL potentiates target therapy in preclinical melanoma models
Source: J Exp Clin Cancer Res. 2026 Jan 8;45:37. doi: 10.1186/s13046-025-03635-w (PMC12870073; doi:10.1186/s13046-025-03635-w)
Supplement: Supplementary file 2 — Supplementary Material 2. [file 13046_2025_3635_MOESM2_ESM.pdf]

ORIGINAL WESTER BLOTS

FIG. 1

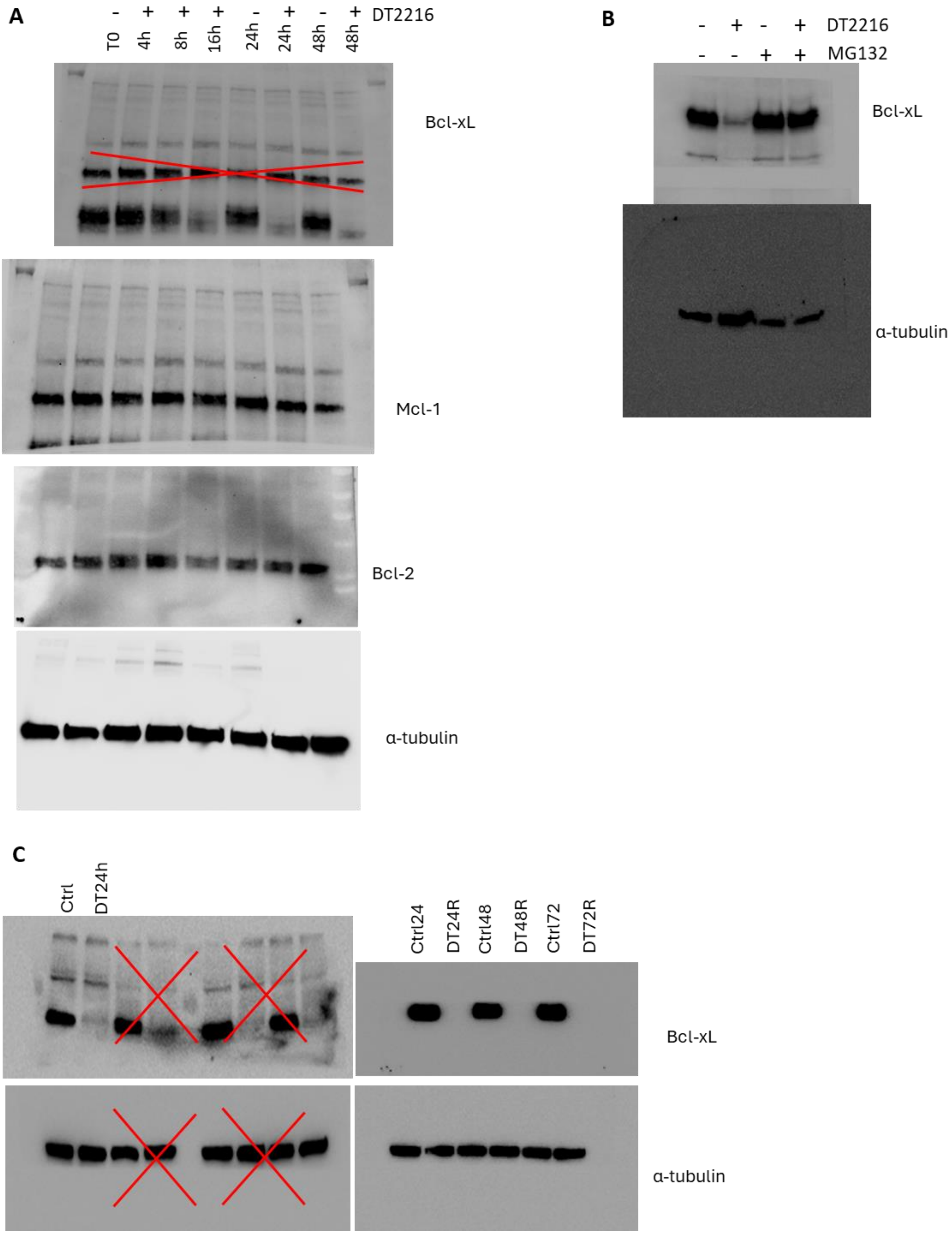

FIG. 1

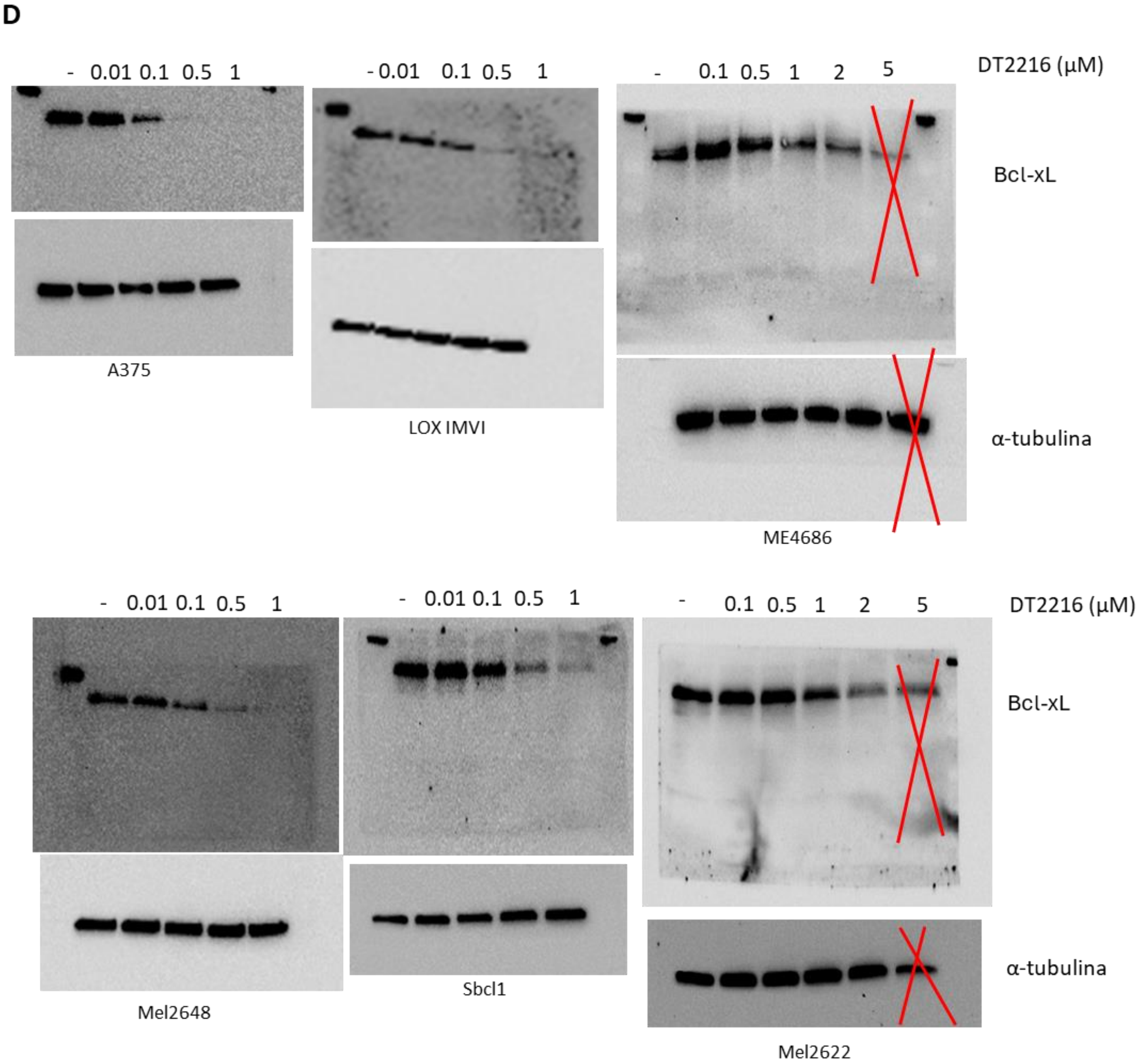
D
- 0.01 0.1 0.5 1
- 0.01 0.1 0.5 1
- 0.1 0.5 1 2 5
DT2216 (µM)
Bcl-xL
A375
LOX IMVI
α-tubulina
ME4686
- 0.01 0.1 0.5 1
- 0.01 0.1 0.5 1
- 0.1 0.5 1 2 5
DT2216 (µM)
Bcl-xL
α-tubulina
Mel2648
Sbcl1
Mel2622

FIG. 4

C

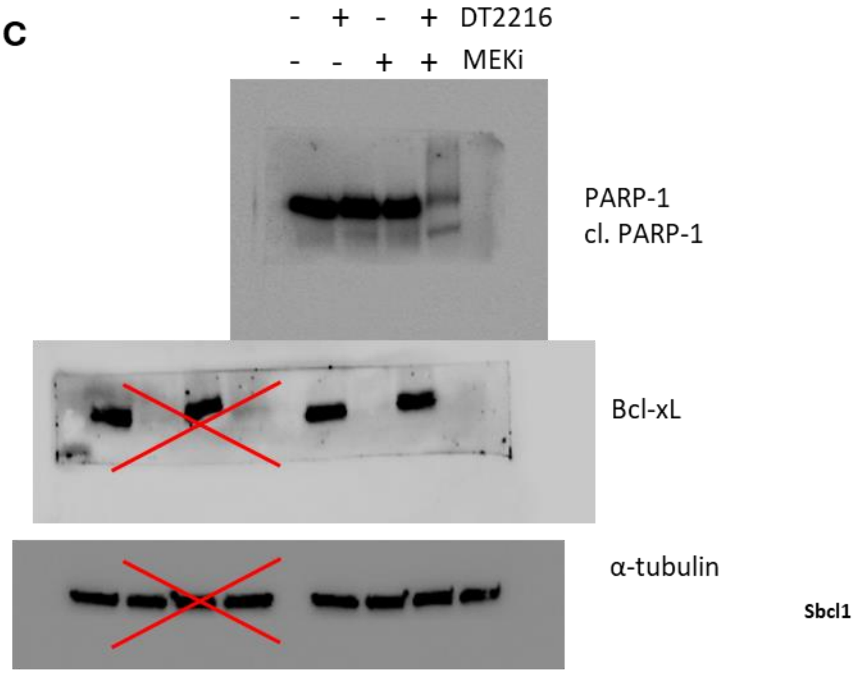

FIG. 5

B

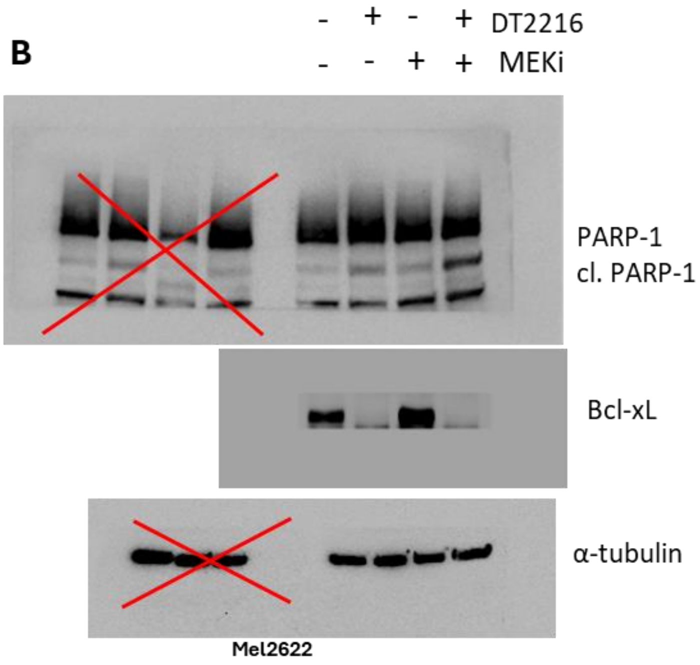

D

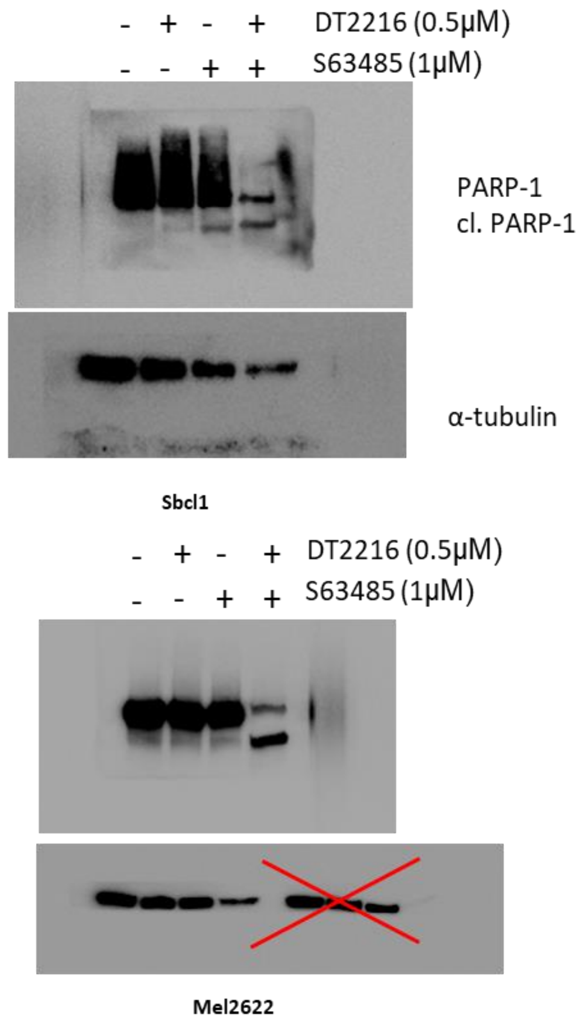

FIG. 7

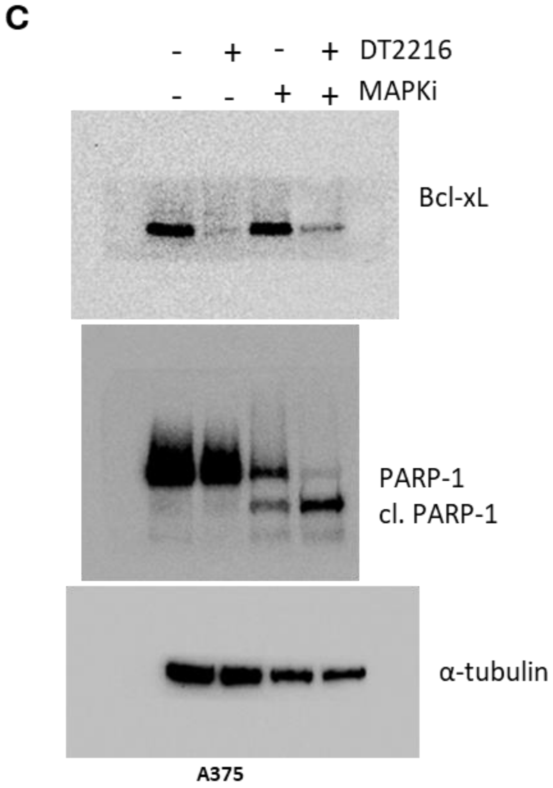

FIG. 8

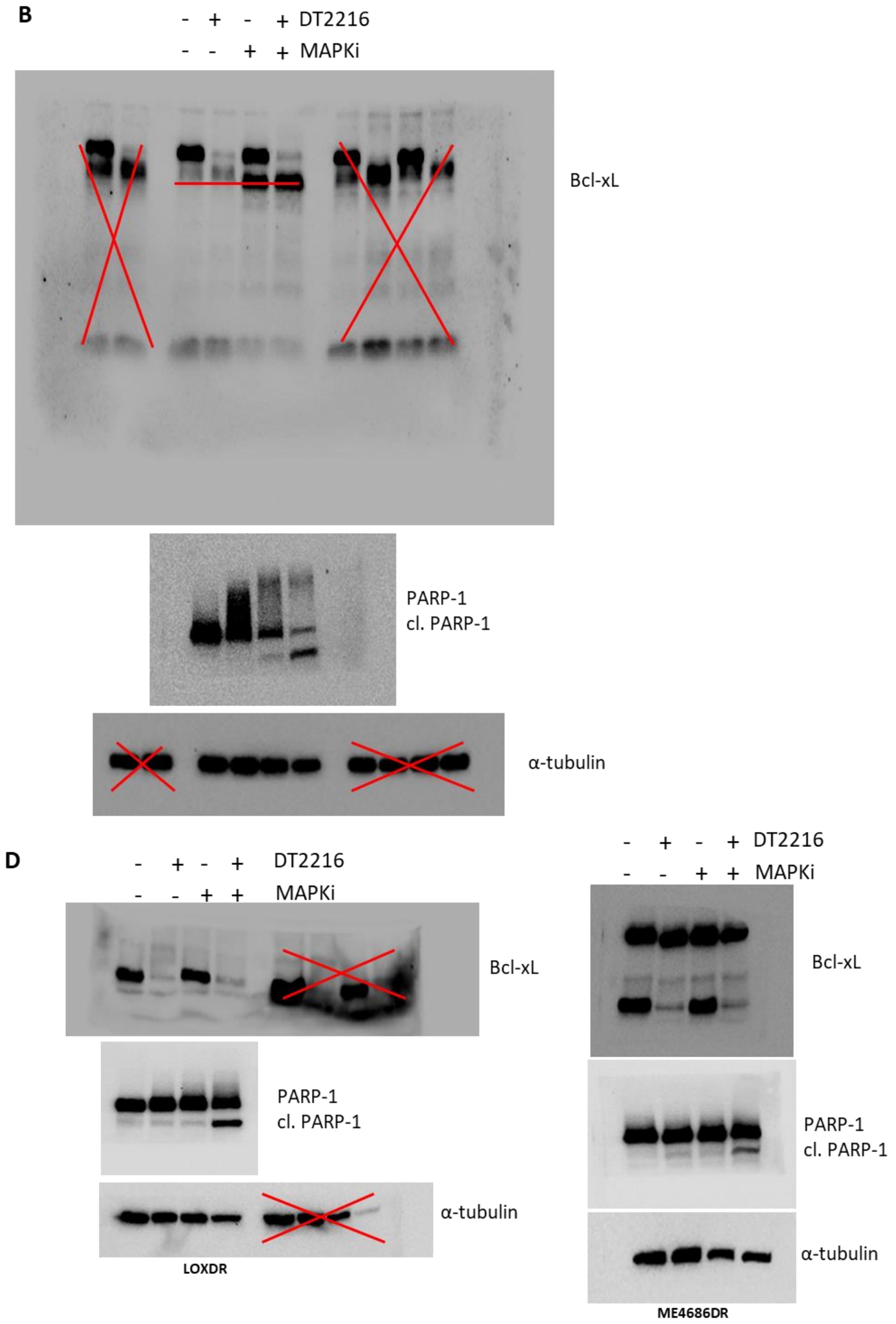

B
- + - + DT2216
- - + + MAPKi
Bcl-xL
PARP-1
cl. PARP-1
α-tubulin
D
- + - + DT2216
- - + + MAPKi
Bcl-xL
PARP-1
cl. PARP-1
α-tubulin
LOXDR
- + - + DT2216
- - + + MAPKi
Bcl-xL
PARP-1
cl. PARP-1
α-tubulin
ME4686DR

Fig.S1

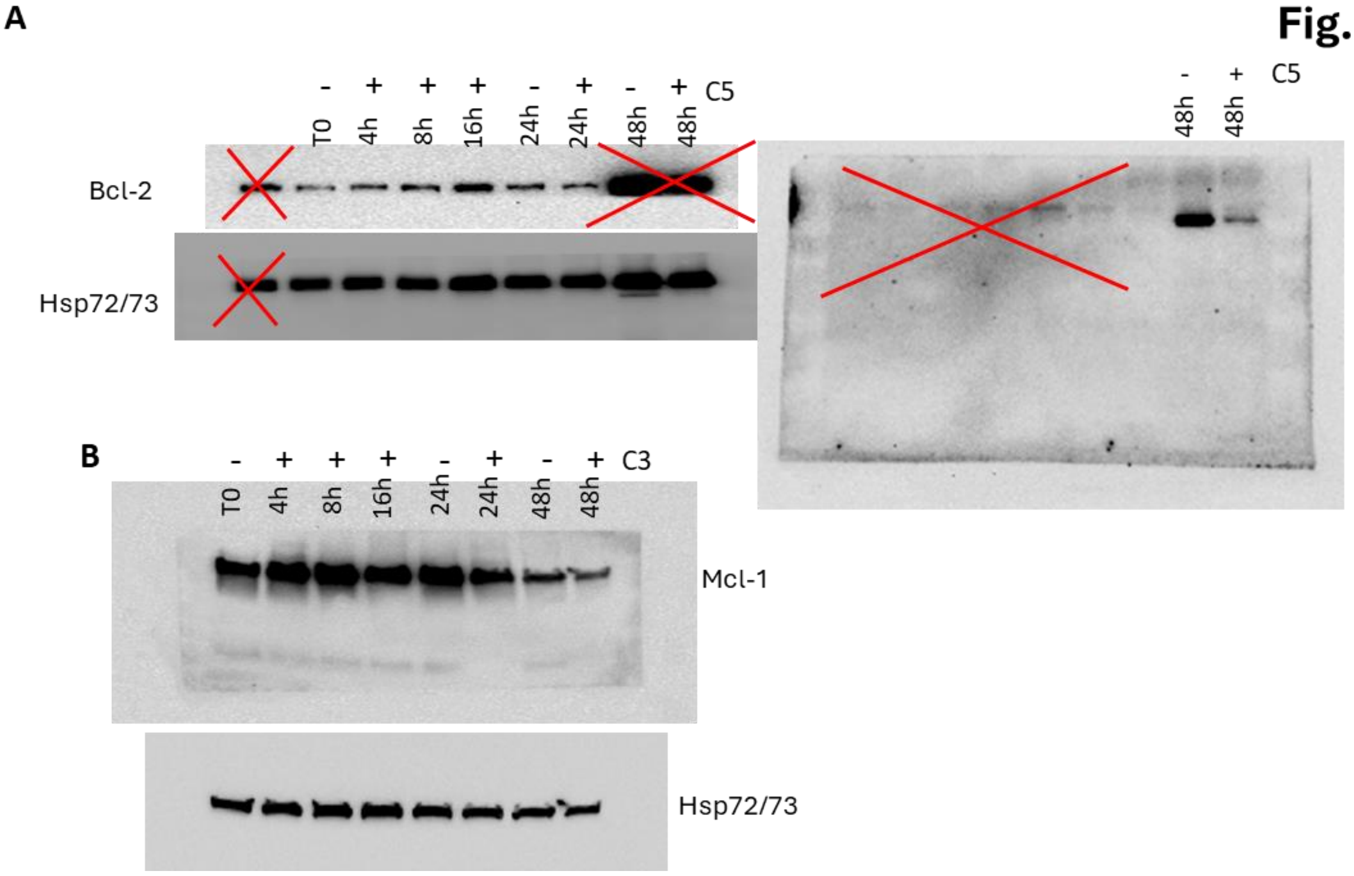
A
- + + + - + - + C5
T0 4h 8h 16h 24h 24h 48h 48h
Bcl-2
Hsp72/73
- + C5
48h 48h
B
- + + + - + - + C3
T0 4h 8h 16h 24h 24h 48h 48h
Mcl-1
Hsp72/73

Fig.S1

C

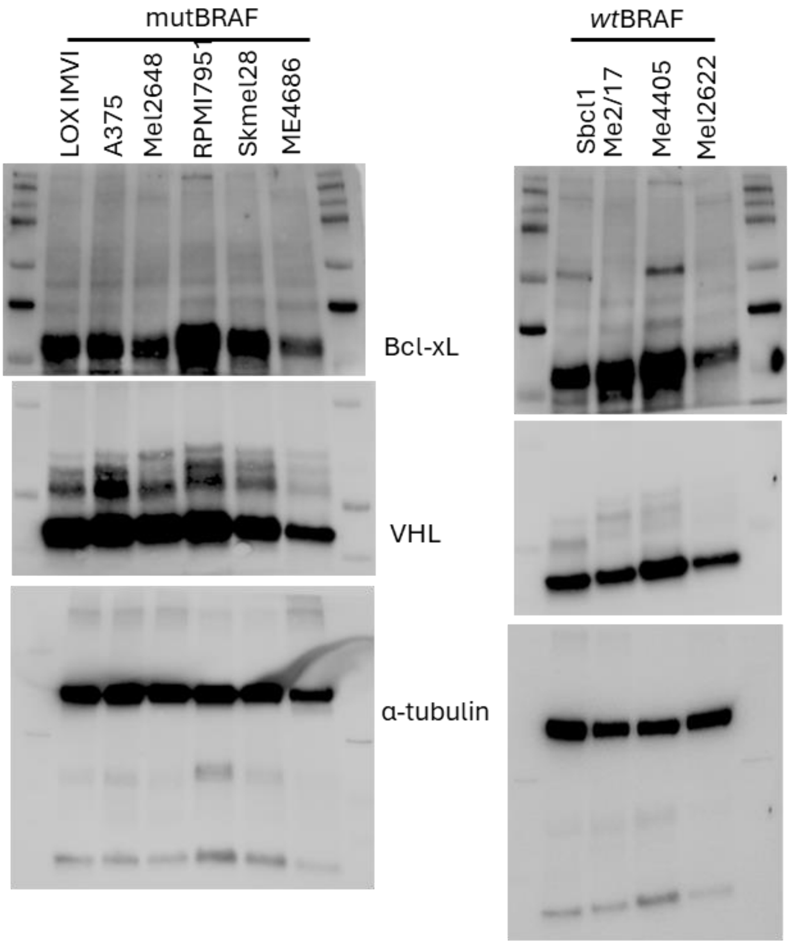
mutBRAF
LOX IMVI
A375
Mel2648
RPMI7951
Skmel28
ME4686
*wt*BRAF
Sbcl1
Me2/17
Me4405
Mel2622
Bcl-xL
VHL
α-tubulin

D

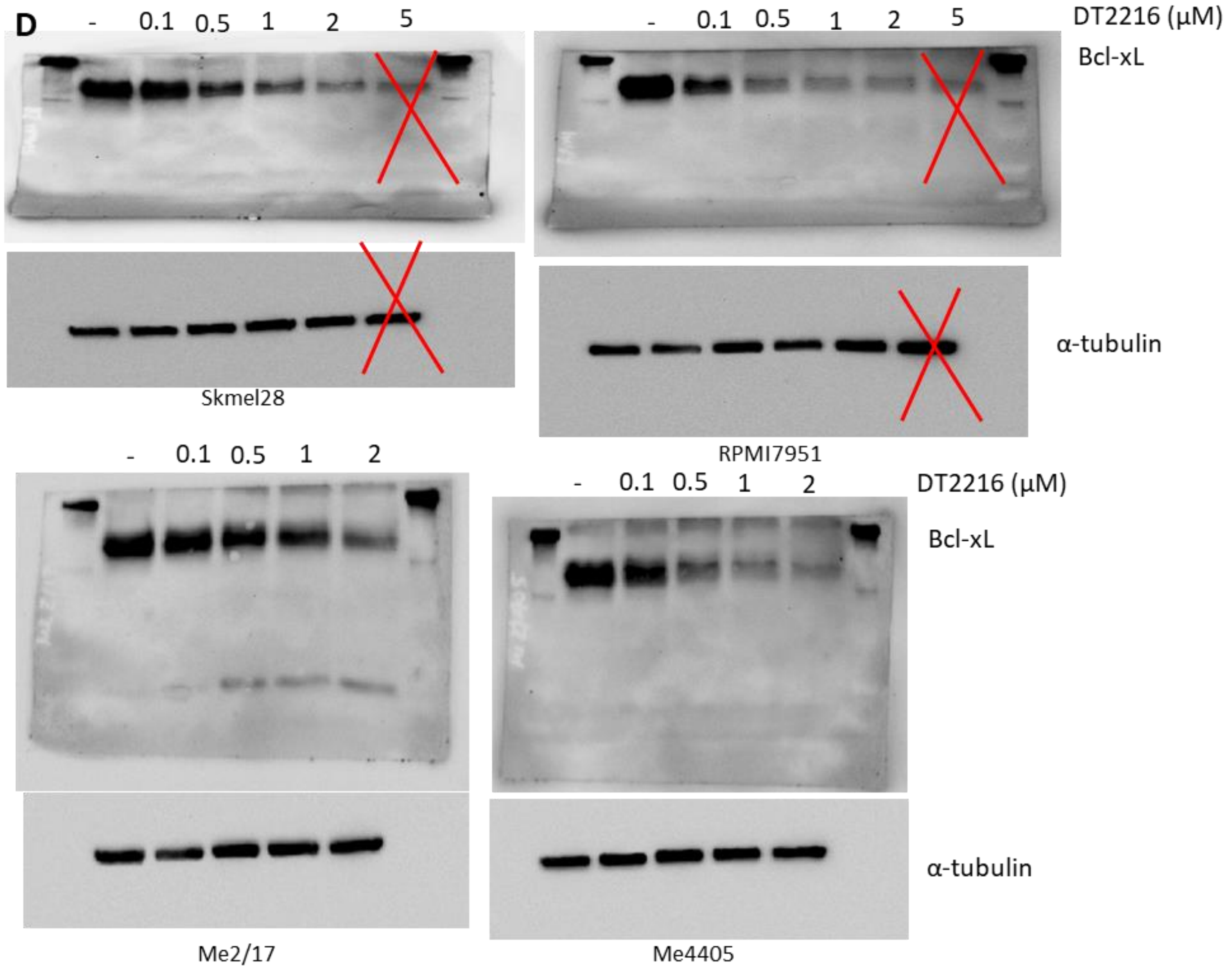
-
0.1
0.5
1
2
5
DT2216 (μM)
Bcl-xL
α-tubulin
Skmel28
RPMI7951
Me2/17
Me4405

Fig.S3

B

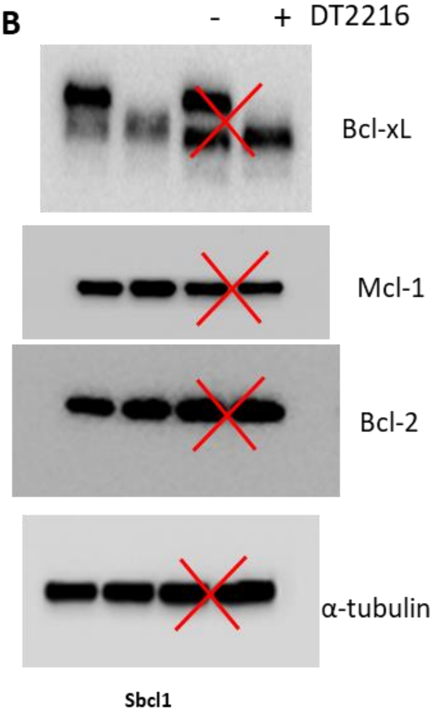

Fig.S4

C

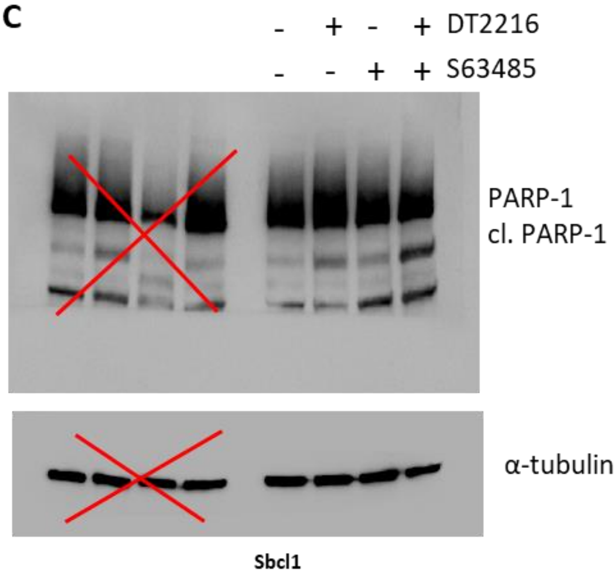

D

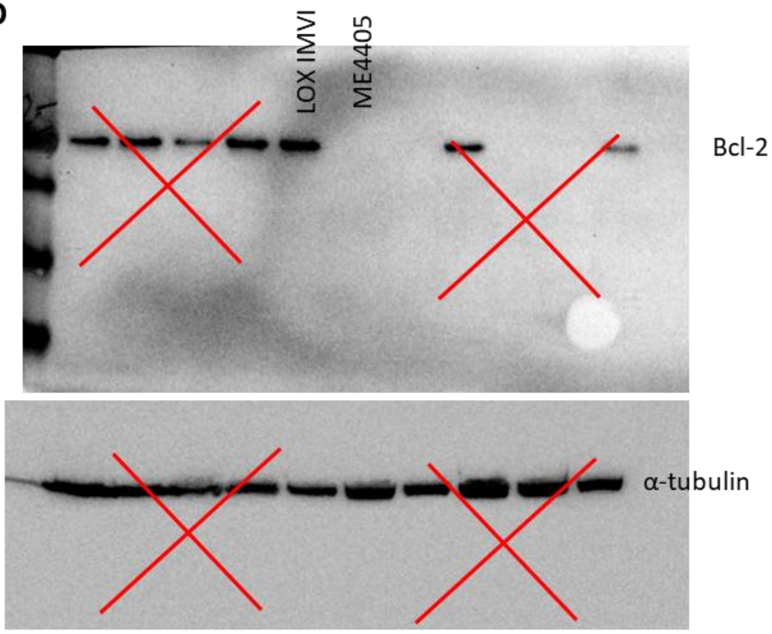

Fig.S5

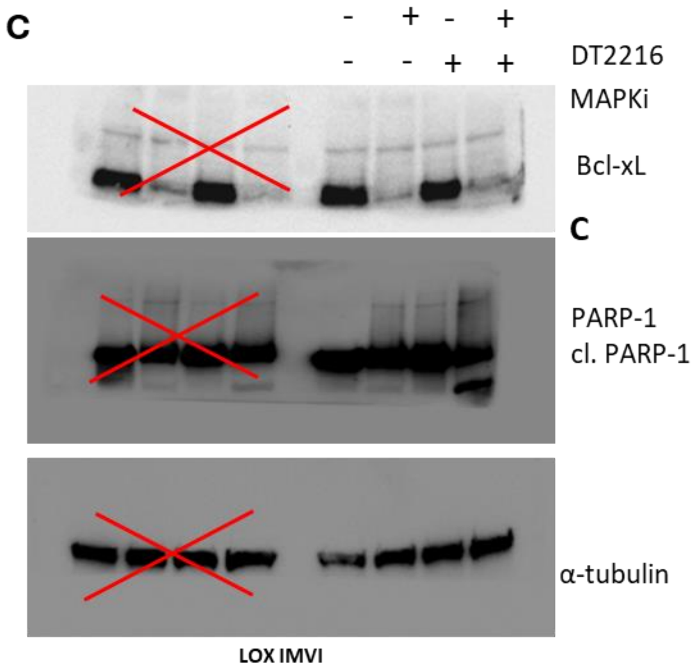
C
- + - +
- - + +
DT2216
MAPKi
Bcl-xL
C
PARP-1
cl. PARP-1
α-tubulin
LOX IMVI

Fig.S6

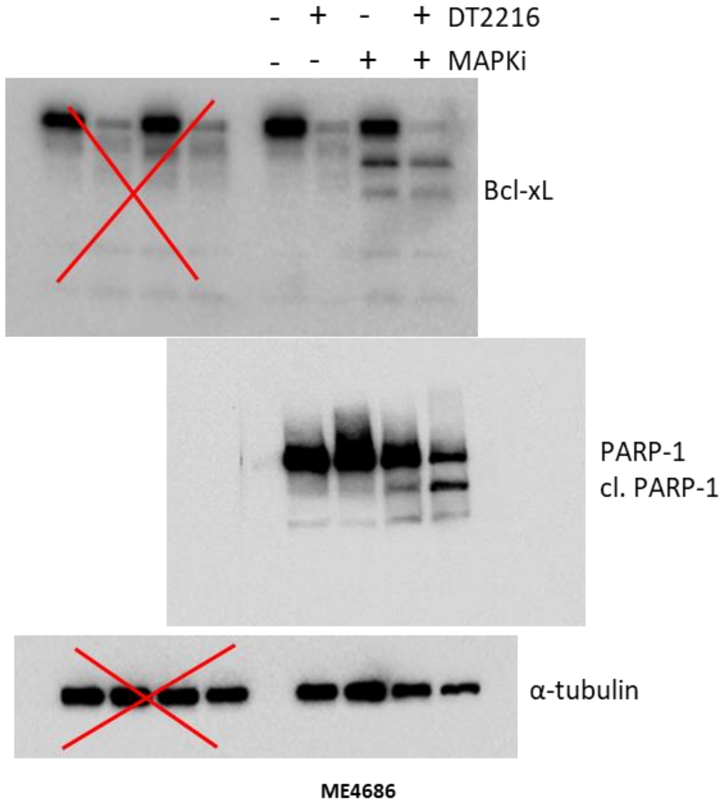
- + - + DT2216
- - + + MAPKi
Bcl-xL
PARP-1
cl. PARP-1
α-tubulin
ME4686
